# Microbial shifts in the aging mouse gut

**DOI:** 10.1186/s40168-014-0050-9

**Published:** 2014-12-05

**Authors:** Morgan GI Langille, Conor J Meehan, Jeremy E Koenig, Akhilesh S Dhanani, Robert A Rose, Susan E Howlett, Robert G Beiko

**Affiliations:** Department of Pharmacology, Dalhousie University, Halifax, Nova Scotia Canada; Faculty of Computer Science, Dalhousie University, Halifax, Nova Scotia Canada; Mycobacteriology Unit, Institute of Tropical Medicine, Antwerp, Belgium; Department of Physiology and Biophysics, Dalhousie University, Halifax, Nova Scotia Canada; Department of Medicine (Geriatric Medicine), Dalhousie University, Halifax, Nova Scotia Canada

**Keywords:** Microbiome, Aging, Mice, B12, *Alistipes*, Frailty index

## Abstract

**Background:**

The changes that occur in the microbiome of aging individuals are unclear, especially in light of the imperfect correlation of frailty with age. Studies in older human subjects have reported subtle effects, but these results may be confounded by other variables that often change with age such as diet and place of residence. To test these associations in a more controlled model system, we examined the relationship between age, frailty, and the gut microbiome of female C57BL/6 J mice.

**Results:**

The frailty index, which is based on the evaluation of 31 clinical signs of deterioration in mice, showed a near-perfect correlation with age. We observed a statistically significant relationship between age and the taxonomic composition of the corresponding microbiome. Consistent with previous human studies, the Rikenellaceae family, which includes the *Alistipes* genus, was the most significantly overrepresented taxon within middle-aged and older mice.

The functional profile of the mouse gut microbiome also varied with host age and frailty. Bacterial-encoded functions that were underrepresented in older mice included cobalamin (B12) and biotin (B7) biosynthesis, and bacterial SOS genes associated with DNA repair. Conversely, creatine degradation, associated with muscle wasting, was overrepresented within the gut microbiomes of the older mice, as were bacterial-encoded β-glucuronidases, which can influence drug-induced epithelial cell toxicity. Older mice also showed an overabundance of monosaccharide utilization genes relative to di-, oligo-, and polysaccharide utilization genes, which may have a substantial impact on gut homeostasis.

**Conclusion:**

We have identified taxonomic and functional patterns that correlate with age and frailty in the mouse microbiome. Differences in functions related to host nutrition and drug pharmacology vary in an age-dependent manner, suggesting that the availability and timing of essential functions may differ significantly with age and frailty. Future work with larger cohorts of mice will aim to separate the effects of age and frailty, and other factors.

**Electronic supplementary material:**

The online version of this article (doi:10.1186/s40168-014-0050-9) contains supplementary material, which is available to authorized users.

## Background

The human microbiome influences and is influenced by several aspects of the host’s health and development [1]. Perturbations to human-associated bacterial communities are associated with many disorders such as colon cancer, autoimmune diseases, inflammatory bowel disease, and *Clostridium difficile* infection [2-8]. The influence of the gut microbiota on human health is driven by interactions between microbes and the host: for example, different groups of bacteria can synthesize energy sources such as butyrate and other short-chain fatty acids [9,10], stimulate the immune system [11,12], and provide protection from pathogens through competitive exclusion and the production of protective compounds such as bacteriocins [13,14].

The age of the host appears to be linked with the composition of the associated microbiome [15,16]. Although many studies have focused on associations between the microbiome and early life stages [15,17,18], relatively few studies have looked into the effect of the microbiome on aging and frailty in later life [19-23]. It is known that the microbiome changes drastically between infant and adult stages of life, with a shift from dominance by *Bifidobacterium* to genera within Bacteroidetes and Clostridia [15,19] that reflects a change from primarily lactate metabolism and plant polysaccharide breakdown to short-chain fatty acid (SCFA) production and vitamin (such as cobalamin/B12) and carbohydrate metabolism. Previous studies have observed changes to the bacterial communities between young/middle-aged adults and older subjects [15,20-22]. Centenarians were found to have a decrease in Clostridia and an increase in Proteobacteria and Bacilli in their gut microbiomes [20]. Such shifts reduce the abundance of SCFA producers and increase the number of facultative anaerobes and opportunistic pathogens (primarily Proteobacteria) within the gut microbiome of the older population [23]. Such shifts would likely lead to increased inflammation, which along with aging-associated changes is a major contributor to the overall frailty of an individual [24]. Studies focusing on the frailty of the host as a factor in microbiome composition found that members of the *Oscillibacter* and *Alistipes* genera were in high abundance in the most frail individuals [22] whereas the abundance of Eubacteriaceae, *Faecalibacterium*, and *Lactobacillus* was reduced [21]. Although these shifts in constituent microbes are likely associated with changes in microbial metabolism and interactions with the host, the consequences of such shifts are not yet understood. Beyond these studies, little is known to date about the link between the microbiome, aging and frailty in terms of perturbations to the microbial communities. Since different microbial taxa often carry out different molecular functions and take on different ecological roles in the gut, we may expect to see commensurate changes in the metabolic potential of the microbes and the manner in which they interact with the host.

Given the potential for the gut microbiome to change as an individual ages, and its possible role in the health of older individuals, our aim is to investigate linkages between aging, frailty, and the microbiome. There are many probable confounding factors in human studies, including change in diet, medications, and housing status (e.g., home residence versus long-term care facilities), making it difficult to identify direct effects of aging and frailty. Although not free of confounding factors themselves, mouse studies allow for better-controlled observations and experiments and have been shown to serve as good models of the human microbiome [25,26]. A frailty model has recently been demonstrated to provide similar information about aging mice [27] as traditional frailty assessments do for older human patients [28]. A previous study of the gut microbiome in older mice related dietary intervention to shifts in bacterial composition [29] but did not investigate changes in relation to age itself. Using metagenomic sequencing of fecal samples, we find shifts both in microbial composition and specific molecular functions that correlate with the age and frailty of the host. Our results corroborate previous findings of microbiome shifts in aging adults and identify changes in taxonomy and function with relevance to aging and frailty.

## Results and discussion

### Murine age groupings and frailty index scores

**Figure 1:**
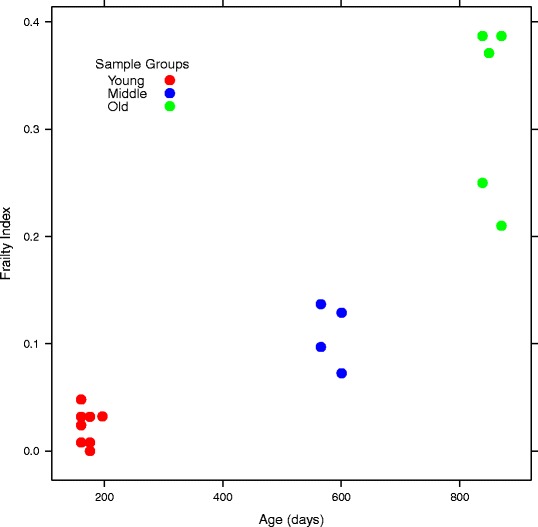
**Age (x-axis) and frailty index (y-axis) for the mice used in this study.** Age correlated significantly with frailty index (Spearman correlation = 0.86, *p* = 1.064 × 10^−5^) and samples were derived from three age groupings: young (red), middle (blue), and old (green). Note that corresponding frailty scores were not performed for 4 of the 21 stool samples, so only 17 points are shown in the plot.

A total of 21 stool samples for metagenomic analysis were collected from ten different mice with varying ages and murine frailty index (FI) scores (Additional file 1). Age correlated with frailty (Spearman correlation = 0.86, *p* = 1.064 × 10^−5^), and fell into three natural groupings which we refer to as ‘young’ (age in days: mean 174 ± s.d. 15; FI: 0.024 ± 0.016; samples = 9; mice = 5), ‘middle’ (age in days: 589 ± 18; FI: 0.097 ± 0.030; samples = 6; mice = 2), and ‘old’ (age in days: 857 ± 16; FI: 0.302 ± 0.088, samples = 6; mice = 3) (Figure 1, Additional file 2). The strong correlation between FI and age reinforces that both are suitable measurements to compare with changes in the gut microbiome. Fares and Howlett showed that a 50% mortality rate occurs around 24 months in mice, which corresponds roughly to age 85 in humans [30]. Our study did not reveal a strong indication that either FI or age was a better predictor of microbiome shifts due to the sample size of the study and the lack of observed variation in FI with respect to age in this particular subset of mice.

### Taxonomic differences between young, middle, and old mouse groups

**Figure 2:**
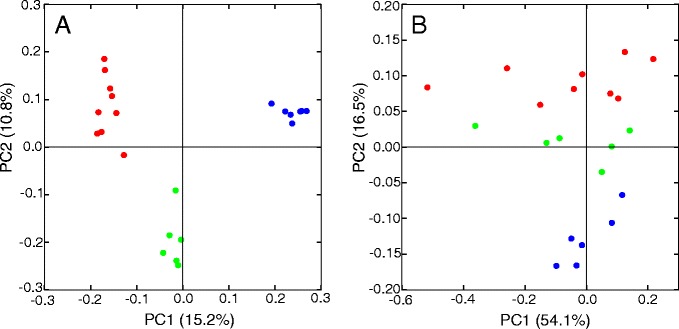
**Principal coordinate analysis of 16S sequences from 21 samples using unweighted (A) and weighted (B) UniFrac shows distinct separation of samples based on their age and frailty into groups of young (red), middle (blue), and old (green).**

To determine if there are differences in the microbiomes of mice of different ages, we compared the taxonomic composition of the microbiome samples using several different methods. Initially, we extracted 16S rRNA ribosomal genes from the metagenomic sequences and assigned these to 97% operational taxonomic units (OTUs) from GreenGenes using the Quantitative Insights Into Microbial Ecology (QIIME) closed-reference OTU-picking protocol. Principal coordinate analysis of the samples showed significant separation by age groups using both weighted UniFrac (ANOSIM *p* value = 0.004) and unweighted UniFrac (ANOSIM *p* = 0.001) (Figure 2). Principal coordinate 1 (PC1) (percent variation explained: 15.2%) of the unweighted UniFrac separates all three sets of samples, while PC2 (10.8%) separates the old group from the other samples even further. In the weighted UniFrac plot, separation of the samples by age only appears after introducing PC2 (16.5%) and not with PC1 (54%), which appears to be driven by substantial changes in taxonomic relative abundance in two samples (Y7-Aug15 and 1E-May23). These two samples both have reduced levels of Bacteroidales relative to Clostridiales and vary greatly from all other samples including those taken from the same mouse within very close time spans (Y7-Aug15: 1 day and 1E-May23: 32 days). In spite of these differences, the clustering of samples by age group even for these two outliers by unweighted UniFrac suggests a strong relationship in qualitative terms. The clearer delineation of samples by age in the unweighted UniFrac analysis suggests that the presence or absence of particular OTUs is more important than the abundance of these OTUs in separating the samples by age. To further test if separation of samples was observed regardless of method, a PCA plot was created that used only the raw abundances of each OTU and did not use a beta diversity measure to relate the OTUs to each other. These results were consistent with our initial PCoA analysis as illustrated by the clear separation of the samples by age group when PC1 (40.0%) and PC2 (23.4%) were plotted (Additional file 3).

The second approach for determining taxonomic composition within the metagenomic samples used 40 conserved protein-coding genes that have previously been described [31] and were assigned using the PhyloSift package [32]. Metagenomic reads matching to each of the conserved protein-coding genes were then inserted into their corresponding reference phylogenetic gene tree using Pplacer [33] and then compared using Pplacer’s edge principal component analysis (edge PCA) [34]. The edge PCA method transforms the placed reads such that each edge in the tree becomes a variable of interest, which is then used for PCA. This has the added advantage of relating each of the principal components directly to the phylogenetic edges that are contributing to that signal. Similar to the weighted UniFrac PCoA, the first principal component (87.4%) from the edge PCA did not provide any separation of the samples, and after visualizing the drivers of PC1 on the phylogenetic tree provided by Pplacer, it seems to be driven by a trade-off between Firmicutes/Clostridia and Bacteroidetes [35]. However, separation of samples by age did appear when using PC2 (6%) and PC3 (4%) (Additional file 4A). Separation between the old and middle samples occurred primarily by PC2 of the edge PCA plot and was driven by the middle group having more Rikenellaceae, Lactobacillaceae, Mycoplasmataceae, and Erysipelotrichaceae, and less Lachnospiraceae, Clostridiaceae, Ruminococcaceae, Prevotellaceae, and Porphyromonadaceae (Additional file 4B). PC3 primarily separated the young samples from the middle and old samples and was driven by the young having more Lactobacillaceae, Prevotellaceae, and Porphyromonadaceae and the middle and old groups having more Rikenellaceae, Lachnospiraceae, Ruminococcaceae, and Clostridiaceae (Additional file 4C). In all cases, the samples of young and old mice were more similar to each other than either was to the middle group. This observation agrees with studies in humans that also showed that middle-aged people had more distinct taxonomic [20] and functional [36] compositions then other adult age groups.

Some samples were taken from the same mouse over a period of time, raising the question of whether intra-mouse correlations artificially increased the apparent similarity within age groups. To address this question, we examined the average beta diversity between all samples taken from the same mouse in a given age group, versus all pairs of samples taken from different mice. We found no significant difference in means (Welch *t* test *p* < 0.05) between the two types of samples at all ages, for both weighted and unweighted UniFrac (Additional file 5). The average distances being compared never differed by more than 0.029 (middle mouse group; unweighted UniFrac), and in the young group, the samples from the same mice had greater beta diversity than those taken from different mice. This lack of difference suggests that there are substantial changes in the microbiome composition over short periods of time but that there is commonality in the microbiome as mice age under controlled laboratory conditions.

**Figure 3:**
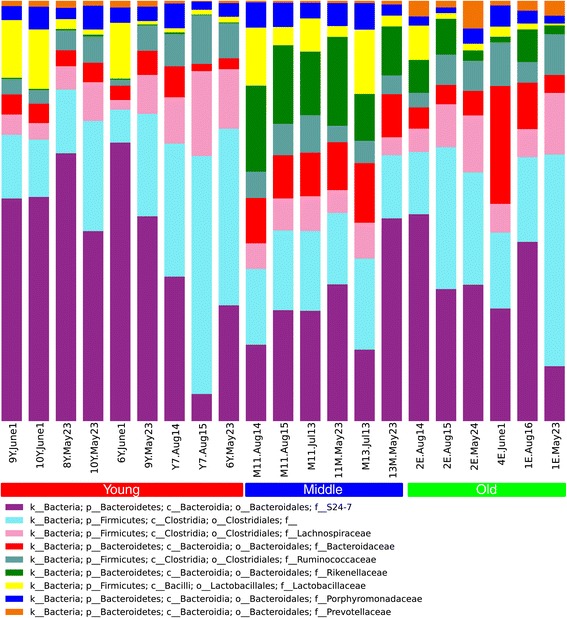
**Taxonomic composition for the nine most abundant families determined using identified 16S sequences, across all 21 samples ordered by increasing age; increasing frailty in each group is ordered from left to right.** For visual clarity, only the nine most abundant families are shown in the plot. Taxonomic ranks are indicated as follows: ‘k’, kingdom (or domain); ‘p’, phylum; ‘c’, class; ‘o’, order; ‘f’, family. The unspecified ‘f__’ represents those OTUs that do not have a specific family name but are known to be within the order Clostridiales.

**Figure 4:**
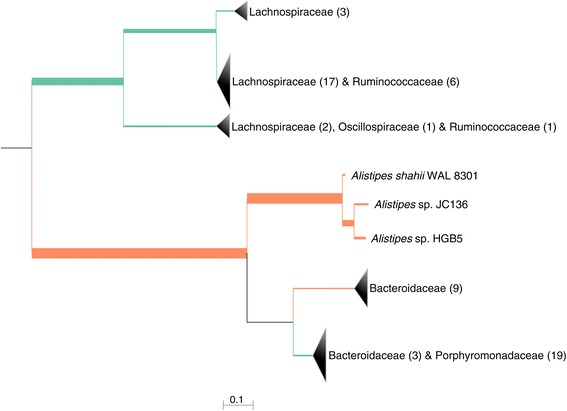
**Taxonomic differences between sample types as predicted by PhyloSift.** Lineages that are overrepresented in the old and middle groups are shown in orange, while those more prevalent in the young group are shown in green. Branches which have no difference in abundance between groups were pruned from the tree. Taxon branches are collapsed to family level where the significant overrepresentation is not to a specific species. Numbers within brackets are the count of taxa within that family.

Taxonomic differences were compared across the different age groups to determine if particular taxa are associated with the gut microbiomes of aging and frail mice. The Rikenellaceae family, which contains the *Alistipes* genus and has previously been linked to microbiomes from elderly people [22], was the most significantly overrepresented family within the middle and old groups in comparison to young mice when using 16S data (Kruskal-Wallis *H* test, Benjamini-Hochberg FDR multiple test correction *p* = 0.007) (Figure 3; Additional files 6 and 7). To ensure that this taxonomic link between frailer mice and people was not an artifact of using the 16S rRNA gene as a marker, an independent analysis using protein markers (PhyloSift) and phylogenetic placement (Pplacer) was conducted. The latter method has the advantage of identifying the phylogenetic context of sequenced reads but does not provide statistical significance testing. However, the results from this method did provide additional support that the *Alistipes* were more abundant within the old and middle mouse groups (Figure 4). The PhyloSift approach indicated that the old mice have lower abundances of organisms from the Lachnospiraceae family, a group often associated with the production of beneficial SCFAs [37], even though the 16S-based method did not show a significant difference (*p* = 0.7) between the age groups. This contrasting result suggests that the type of phylogenetic marker used for taxonomic assessment can give conflicting results for some taxon families. Lastly, the PhyloSift approach indicated that some members of the Bacteroidaceae family were abundant in the old and middle groups, while fewer were abundant in the young group, which did agree with the 16S results (old: 9.2% ± 7.9%; middle: 10.3% ± 1.3%; young: 3.9% ± 1.4%, *p* = 0.06).

Previous research in humans showed a negative relationship between frailty and the abundance of Eubacteriaceae, *Faecalibacterium*, and *Lactobacillus* [21]. We found virtually no Eubacteriaceae (eight sequences total) within any of our samples and no difference in proportions of *Faecalibacterium* between the age groups (*p* = 0.828; Additional file 6). These differing results could be due to unique differences between aging in mice and in humans or due to confounding signals from diet or habitat change in the elderly study. Although a recent finding did identify in aging mice the same decrease in *Lactobacillus* as in the elderly [38], no significant decrease in *Lactobacillus* was observed (old: 2.4% ± 2.4%; middle: 7.0% ± 4.6%; young: 4.9% ± 5.3%, *p* = 0.44; Additional file 6). Zhang et al. also reported a large phylum shift for all their aged mice from Firmicutes to Bacteroidetes, but we did not observe any change in the Firmicutes/Bacteroidetes ratio among our different age groups [38]. Additionally, we noted that the genus *Akkermansia* was nearly absent from the old group (0.003% ± 0.004%), compared to the middle (0.39% ± 0.27%) and young mice (0.50% ± 0.8%), but the difference in means was again not significant at an alpha threshold of 0.05 (*p* = 0.058; Additional file 6). *Akkermansia* has been linked to healthy microbiomes in some studies [39,40], although members of this genus may also exacerbate certain types of infection via mucin degradation [41].

### Functional differences among microbiomes

Annotation of metagenomic sequences using MG-RAST led to the identification of 99 SEED level 3 categories that had significant mean abundance differences across the young, middle, and old categories using STAMP [42] (Kruskal-Wallis *p* < 0.05 with Benjamini-Hochberg FDR correction). Detailed results are shown in Additional file 8; here, we focus on functions of particular interest due to their possible roles in aging and frailty.

#### Carbohydrate and lactate utilization

**Figure 5:**
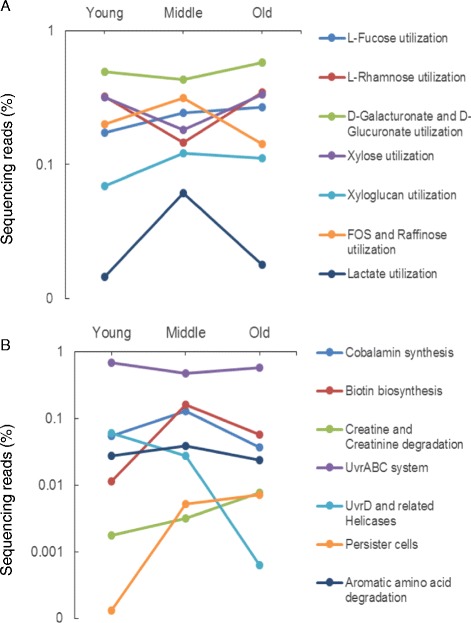
**Notable functional categories with significant differences in abundance across the young, middle, and old mice. (A)** Functions related to carbohydrate and lactate metabolism. **(B)** Other functions including vitamin biosynthesis and DNA repair.

Most monosaccharide utilization categories were overrepresented in the old group compared to the young and middle groups, with the complex polysaccharide-xyloglucan utilization category underrepresented in the old group (0.112% ± 0.014%, *p* = 0.034) compared to the middle group (0.122% ± 0.016%) (Figure 5A). Monosaccharide utilization categories with statistically significant changes among age groups were L-fucose (old: 0.268% ± 0.015%; middle: 0.243% ± 0.015%; young: 0.172% ± 0.022%, *p* = 0.034), L-rhamnose (old: 0.347% ± 0.059%; middle: 0.146% ± 0.014%; young: 0.322% ± 0.064%, *p* = 0.040), D-galacturonate and D-glucuronate (old: 0.579% ± 0.032%; middle: 0.431% ± 0.052%; young: 0.492% ± 0.025%, *p* = 0.036), and xylose (old: 0.333% ± 0.040%; middle: 0.182% ± 0.040%; young: 0.317% ± 0.055%, *p* = 0.042). This observation agrees with earlier reports of decline in the saccharolytic potential of microbiome with age and further suggests a limited supply of simple sugars for gut epithelium function in the old age group.

Additionally, the D-galacturonate and D-glucuronate utilization category includes glucuronidases that catalyze the hydrolysis of D-glucuronic acid as a part of complex carbohydrate metabolism [43]. Elevated levels of human β-glucuronidase activity in tumors compared to normal cells led to the development of several selective cancer chemotherapeutics [44]. However, many microbial species contain β-glucuronidase homologs, which have been shown to reactivate the cancer drug irinotecan (e.g., CPT-11) leading to severe diarrhea and thus limiting the efficacy of the drug [45,46]. Fructooligosaccharides (FOS) and raffinose are carbohydrates that are commonly used as prebiotics to promote growth of Lactobacilli and Bifidobacteria in the GI tract of older individuals [29,47]. The underrepresentation of FOS and raffinose utilization in old mice (0.142% ± 0.037%, *p* = 0.031) compared to mice in the young (0.200% ± 0.044%) and middle (0.314% ± 0.048%) groups could be another contributing factor to perturbing beneficial microbe populations and an increased risk of opportunistic infection.

The concentration of lactate in the colon is typically maintained by lactate-utilizing bacteria and metabolism of D-lactate by host lactases [48]. The reduced colonic ability to utilize lactate in the old group (0.018% ± 0.011%, *p* = 0.041) compared to the middle group (0.061% ± 0.014%) and the negative effect of aging on host lactase activity [49] can adversely affect host health with accumulation of lactate in the colon. High fecal lactate is associated with ulcerative colitis and other inflammatory bowel diseases [50,51].

#### Biosynthesis of vitamins and creatine degradation

Cobalamin (B12) biosynthesis was significantly underrepresented within the old group (0.037% ± 0.041%, *p* = 0.041) compared to the middle (0.129% ± 0.026%) and young (0.054% ± 0.018%) groups (Figure 5B). In a previous study, 55% of people aged 43 and under had a microbiome encoding a gene, *cbiN* (cobalamin biosynthesis protein; pfam ID PF02553), involved in B12 biosynthesis, while only 11% those over the age of 43 had a copy of it [36]. Deficiency in B12 within elderly people has been well documented in the past [52,53], and the decreased potential of the microbiome to synthesize vitamin B12 has been suggested as a contributing factor [54]. Conversely, creatine and creatinine degradation functionality was overrepresented in the gut microbiomes of the old group (0.0076% ± 0.002%, *p* = 0.030) compared to the middle (0.0031% ± 0.001%) and young (0.0018% ± 0.001%) groups. Creatine supplementation has been shown to promote muscle strength and hypertrophy in elderly people [55]. Therefore, an increase in degradation of creatine by the microbiome may have negative consequences for the host if creatine abundance was limited and thus indirectly affected the FI by leading to reduced lean mass.

Another vitamin, biotin (vitamin B7), is synthesized in considerable amounts by microbiota of the large intestine (sometimes greater than the amount taken in the diet), and a significant portion of this is absorbed by human and animal colonocytes [56]. Biotin synthesis was significantly underrepresented within the young (0.011% ± 0.022%, *p* = 0.040) and old (0.058% ± 0.103%) groups compared to the middle group (0.158% ± 0.044%). Younger individuals require less biotin compared to those of middle and old age, so low contributions from gut microbiota in the young group may not lead to biotin deficiencies under a normal diet. In the old group, however, low biotin can lead to biotin deficiency which in turn increases colon cancer risk [57].

#### DNA repair and persister cells

Bacterial SOS genes (including *uvrABC* and *uvrD*) play an essential function in bacterial nucleotide excision repair and decreasing the rate of mutagenesis [58]. Although no observations have been made until now on the status of DNA repair in any microbiome studies, the significant underrepresentation of *uvrD* (0.0006% ± 0.0008%, *p* = 0.040) and *uvrABC* (0.57% ± 0.10%, *p* = 0.039) within the old group compared to the young group (*uvrD*: 0.061% ± 0.029%; *uvrABC*: 0.675% ± 0.066%) suggests potentially higher rates of mutagenesis and the possibility of acquiring antibiotic resistance within the gut microbiota of the old group. The rise in functional groups associated with persister cells from young (undetectable, *p* = 0.034) and middle (0.005% ± 0.002%) groups to the old group (0.007% ± 0.004%) points to the possibility of multidrug-resistant bacterial populations which may contribute to inflammation in old age [20].

#### Amino acid degradation

Rampelli et al. [23] suggested an association of aromatic amino acid degradation (except histidine) with aging. In the present study, there was no change in tryptophan and histidine metabolism among age groups. However, we found significant underrepresentation of other aromatic amino acid degradation within the old group (0.024% ± 0.003%, *p* = 0.049) compared to young (0.028% ± 0.004%) and middle (0.039% ± 0.006%) groups, although the effect size was very small.

#### Taxonomic assignments of identified functions

Using the RITA software package [59], we performed taxonomic assignment of sequence reads to determine if the shift in abundance was accompanied by a shift of a specific set of microbes. Many key functions showed a shift from genera *Akkermansia* and *Parabacteroides* to *Bacteroides* and Firmicutes. For example, 49.5% of cobalamin synthesis genes in the young and middle mouse groups were assigned to the genus *Bacteroides*, versus 71.1% in the old mice. Conversely, the contribution of *Parabacteroides* to this function dropped from 24.8% to 7.0%. *Akkermansia* was the predicted source of 74.4% of UvrD and related functional genes in the young and middle mouse groups, versus 13.3% in the old mouse cohort, which is instead dominated by *Bacteroides* and to a lesser extent *Oscillibacter* and *Clostridium*. Although the assignment of functions to taxonomic groups can be error-prone, the difference in these assignments between samples from mice of different ages indicates that differences exist, even if some assignments may be suspect.

## Conclusions

Although previous microbiome studies in humans have shown changes with age, the results were confounded by other possible impacts on the microbiome such as diet, living conditions, and medications that also change with age. Here, we have used a mouse model that allows us to control for these factors, and we have observed taxonomic and functional changes in the microbiome that correlate with age and frailty. Consistent with an earlier study of frail humans, the genus *Alistipes* was found to be overrepresented in old mice, suggesting there may be some parallel shifts that occur in aging human and mouse populations. Further, we identify several additional bacterial taxa and functions that may be associated with the aging process. Our results suggest that the aging microbiome could have an effect on the availability of vitamins (B12 and B7) and creatine, DNA repair, and carbohydrate metabolism as well as the potential to interfere with some drug treatments.

Further work with larger mouse cohorts, particularly older mice with a range of frailty scores, will be needed to separate the effects of frailty and aging on the microbiome. Larger longitudinal studies would also clarify the relationship between specific clinical attributes and changes to the microbiome during aging. For example, previous work showed a steady increase in mouse weight up to a peak at 15–20 months, followed by a steady decline as the mice age [60]. The microbiomes of obese individuals tend to have different taxonomic and metabolic properties [61]; there may be corresponding weight-associated microbiome shifts as mice age. Another important factor within this study is the possible role of cage effects on the microbiome. Hildebrand et al. showed that up to 30% of the variation in the microbiome can arise from cage effects, possibly due to coprophagy [62], and the authors recommended that groups of interest be housed in the same cage, or individually. However, neither of these approaches is appropriate since solitary cages for the entire life of a mouse can induce high levels of stress [63] which may in turn influence the microbiome, while a single cage for all mice is not practical for large-scale animal studies. Explicit tests of housing strategies are needed to examine the effects of this important factor. Although larger studies will be necessary to reveal the fine details of aging, frailty, and the microbiome, our results show that the mouse microbiome changes throughout various life stages and suggests that similar changes in humans may have a significant effect on health.

## Methods

### Subject mice and frailty assessment

Female C57BL/6 J mice purchased from Charles River (St. Constant, Quebec) at 3–4 weeks of age were housed in micro-isolator cages on a 12-h light/dark cycle in the Carlton Animal Care Facility at Dalhousie University. All mice in this study had free access to the same food (Prolab RMH 3000, LabDiet, St. Louis, MO) and water in their home cages. Three ages of mice were used in the experiments: a young adult group (174 ± 15 days old), a middle adult group (589 ± 18 days old), and old adult group (857 ± 16 days old). Study animals from the same age group were all housed in the same cage, with the exception of one old mouse which was housed together with another old mouse from which no data were obtained. Experiments were performed following the guidelines outlined by the Canadian Council on Animal Care Guide to the Care and Use of Experimental Animals (CCAC, Ottawa, ON: Vol. 1, 2nd edition, 1993; Vol. 2, 1984). All animal protocols were approved by the Dalhousie University Committee on Laboratory Animals.

Clinical examinations were performed between 10 am and 1 pm every day. Animals were first observed in their home cage and then taken to an assessment room. Mice were weighed, and body surface temperature was measured with an infrared temperature probe directed at the abdomen (Infrascan; La Crosse Technology). A brief clinical exam was then used to create a FI score for each mouse as described above.

### Fecal sample collection and sequencing

Each mouse was placed in an empty, sterilized 43-cm-long, 21-cm-wide cage for 1.5 to 2 h to collect individual fecal samples in a manner similar to Lomansey et al. [64]. A duration of 1.5 to 2 h of isolation may induce some stress in the mice, although much longer times seem to be required to induce major changes in behavior consistent with stress [65]. Samples were collected between 10 am and 1 pm. To minimize sample contamination, mice had no access to food and water during this time and sterile forceps were used for sample handling. On average, mice produced between 100 and 200 mg of sample during each collection period. Immediately after collection, samples were frozen at −80°C.

Genomic DNA (gDNA) was isolated from fecal samples using the PowerSoil DNA isolation kit (MO BIO Laboratories, Inc.) according to instructions from the manufacturer. Once the isolation procedure was completed, the concentration of gDNA was determined for each sample by spectrophotometry and the sample was then frozen at −80°C. Samples were then shipped to Génome Québec (McGill University and Génome Québec Innovation Centre, Montreal, Québec, Canada) where they underwent quality control assessment to confirm sample integrity. Metagenomic sequencing of the microbial DNA extracted from the 21 stool samples was conducted on an Illumina HiSeq generating 100-bp paired-end reads. This produced a total of 85.61 Gbps (mean 2.04 ± 0.81 Gbps per sample) and 428.06 million reads (mean 9.96 ± 0.37 million reads per sample).

Raw FASTQ files for each paired end of the 21 samples were processed with MG-RAST [66] and made publicly available (project id: 3907). MG-RAST default pipeline options were used, including dereplication, screening against *M. musculus* NCBI v37 to remove host DNA sequences, and dynamic read trimming for quality control of sequence reads.

### Taxonomic and functional analysis

In this study, three different methods were used to characterize the taxonomic diversity of samples: (i) using only the 16S rRNA gene (16S), (ii) using 40 different conserved protein genes [31], and (iii) assigning taxonomic classifications to metagenomic reads. Each of these methods is described in more detail below.

Files containing ribosomal 16S rRNA gene (16S) fragments as annotated by MG-RAST were downloaded for each paired end (e.g., ‘425.search.rna.fna’) and combined into their respective sample for further processing with QIIME [67]. The putative 16S reads were assigned to existing OTUs using a closed picking protocol that considers only OTUs already present in a reference database. We used the QIIME-compatible UCLUST version 1.2.22q [68] to map the reads to version 13_5 reference package of GreenGenes [69] using a 97% identity threshold. Every sample was rarefied to 15,000 OTUs to remove possible bias from the variation in sequencing depth for different samples. To visualize and compare the differences between samples, principal coordinate analysis (PCoA) plots were generated with weighted and unweighted UniFrac beta diversity metrics [70]. In addition, a PCA plot based only on the relative OTU abundances in each sample was generated using the R function ‘prcomp’. Taxonomic summary bar charts were created for 16S data by collapsing to different taxonomic ranks (level 5) using QIIME’s ‘summarize_taxa.py’ script, taking only the top ten most abundant families across all samples, renormalizing by sum samples, and then plotting using QIIME’s ‘plot_taxa_summary.py’ Python script.

PhyloSift was used as an alternative method to 16S for determining taxonomic composition [32]. PhyloSift uses 40 conserved protein-coding genes that have previously been used for phylogenomic analysis [31] and places metagenomic read fragments onto a reference tree of sequenced genomes using Pplacer [33]. Metagenomic reads passing MG-RAST quality control were downloaded (‘299.screen.passed.fna’), and paired ends were used as input to PhyloSift (git branch development version: 9a03023cb) with default options. The Pplacer ‘.jplace’ files representing the sequences placed on a concatenated 40-gene tree output by PhyloSift for each sample were used to create an ‘edge PCA’ [34] and visualized in R. The individual sample ‘.jplace’ files were then merged into different age groupings using the ‘guppy merge’ command. Comparison of age groupings was conducted using the pairwise ‘guppy kr_heat’ program which allows visualization of the differences in organism abundance between two groupings on a phylogenetic tree.

Functional SEED [71] annotations including abundance information and actual annotated sequences were downloaded and formatted from the MG-RAST API using in-house scripts that are available at Github (https://github.com/mlangill/get_mgrast_data). Sequences with functions that statistically varied over the age groupings were filtered; a subset of these that yielded reliable sequence counts were subjected to taxonomic analysis. To determine and compare the taxonomic characterization of these particular functions, metagenomic reads annotated with these functions were analyzed using RITA [59]. Briefly, RITA uses a reference database of sequenced and draft genomes to list the annotated proteins for each genome and construct compositional models of each genome. RITA uses a combination of homology search and compositional analysis to assign sequences to different classification groups. We used RITA v1.0.1 with a database of 2,987 finished and draft genomes, with USEARCH version 4.1.93 [68] used for the homology search and FCP version 1.0.3 [72] for compositional matching. Classes of RITA results considered for summary purposes were as follows: (i) cases where the top compositional and homology matches agreed with one another at the genus level and (ii) cases where the expectation value of the best-matching genome was at least 10 orders of magnitude better than the best-matching genome from another genus. Summaries were constructed for a small set of taxa that showed variation between a group comprising young and middle mice and old mice: *Parabacteroides*, *Bacteroides*, and *Lactobacillus*; all remaining genera in phyla Bacteroidetes and Firmicutes; and phylum Verrucomicrobia.

### Statistical analysis

The statistical significance of samples between age groupings was calculated using ANOSIM as implemented in the R package vegan version 2.0-10 [73], with 999 permutations and the weighted and unweighted UniFrac distance matrices. All multiple-group comparisons were done using the Kruskal-Wallis *H* test with Benjamini-Hochberg FDR multiple test correction as calculated within STAMP version 2.0.7 [42]. Where appropriate, reported *p* values are those corrected for multiple testing. Abundance values reported in the results and in the additional files are reported as mean relative abundances for the age groups along with the standard deviation.

## Availability of supporting data

The data set supporting the results of this article is available in the MG-RAST repository, as project 3907, http://metagenomics.anl.gov/linkin.cgi?project=3907.

## Additional files

Additional file 1:
**Clinical measurement components of murine frailty index scores.** Individual frailty index scores were obtained for each mouse with a non-invasive clinical frailty index tool that has been described in detail previously [74]. Clinical assessment of the mice included evaluation of the integument, musculoskeletal system, vestibulocochlear and auditory systems, ocular and nasal systems, digestive system, urogenital system, respiratory system, signs of discomfort, as well as the body weight (g) and body surface temperature (°C). A list of each potential deficit evaluated in this study is shown in the left hand column of the table. Each potential deficit could have a minimum score of 0 (no deficit present) and a maximum score of 1 (severe deficit). For each animal, the scores for each deficit were added and divided by the total number of deficits measured (31) to yield a frailty index score of between 0 and 1. The average score (±SEM) for each potential deficit for all three age groups is also shown in the table. Additional file 2:
**Microbiome sample metadata.** Microbiome metadata for 21 samples from 10 mice. Additional file 3:
**PCA plot of relative OTU abundance.** PCA plot based only on the relative OTU abundances in each sample, generated using the R function ‘prcomp’ shows separation of samples by age groups. Additional file 4:
**Pplacer edge PCA plot.** Taxonomic separation of samples from protein-coding metagenome markers using the PhyloSift and Pplacer packages is shown using an edge PCA plot **(A)** with taxa contributing to the signal shown for PC2 **(B)** and PC3 **(C)**. Taxa contributing to the positive direction of the PC are shown in orange while those contributing in the negative direction of the PC are shown in green. Branches that did not contribute to the PC were pruned from the tree. Taxon branches are collapsed to family level where the overrepresentation inferred by Pplacer is not restricted to a single species. Numbers within brackets are the count of taxa within that family. Additional file 5:
**Variation within samples taken from same and different mice.** Comparison of beta-diversity measurements between samples taken from either the same mouse or a different mouse for each age group (young, middle, old) using weighted UniFrac **(A)** or unweighted UniFrac **(B)**. Additional file 6:
**Taxonomic assignments.** The proportion of sequences assigned to each sample at each taxonomic rank, along with means for each age group and significance of difference in means using Kruskal-Wallis *H* -test with Benjamini-Hochberg FDR multiple test correction, is illustrated. Taxa with *p* value <0.05 are highlighted in green. Additional file 7:
**Taxonomic composition of samples.** Taxonomic composition identified using 16S sequences for all 21 samples ordered by increasing age and frailty from left to right at the phylum **(A)**, family **(B)**, and genus **(C)** levels. Additional file 8:
**Functional assignments.** List of all SEED level 3 category functions with significance of difference in means using Kruskal-Wallis *H* test with Benjamini-Hochberg FDR multiple test correction. SEED level 3 categories with *p* value <0.05 are highlighted in green.

## Abbreviations

gDNA: Genomic DNA
PCA: Principal component analysis
PCoA: Principal coordinate analysis
SCFA: Short-chain fatty acid
