# Supplementary material for: Microbial shifts in the aging mouse gut
Source: Microbiome. 2014 Dec 5;2:50. doi: 10.1186/s40168-014-0050-9 (PMC4269096; doi:10.1186/s40168-014-0050-9)
Supplement: Additional file 1: — Clinical measurement components of murine frailty index scores. Individual frailty index scores were obtained for each mouse with a non-invasive clinical frailty index tool that has been described in detail previously [74]. Clinical assessment of the mice included evaluation of the integument, musculoskeletal system, vestibulocochlear and auditory systems, ocular and nasal systems, digestive system, urogenital system, respiratory system, signs of discomfort, as well as the body weight (g) and body surface temperature (°C). A list of each potential deficit evaluated in this study is shown in the left hand column of the table. Each potential deficit could have a minimum score of 0 (no deficit present) and a maximum score of 1 (severe deficit). For each animal, the scores for each deficit were added and divided by the total number of deficits measured (31) to yield a frailty index score of between 0 and 1. The average score (±SEM) for each potential deficit for all three age groups is also shown in the table. [file 40168_2014_50_MOESM1_ESM.pdf]

Additional File 1: Individual frailty index scores were obtained for each mouse with a non-invasive clinical frailty index tool that has been described in detail previously (Whitehead et al., 2014). Clinical assessment of the mice included evaluation of the integument, musculoskeletal system, vestibulocochlear and auditory systems, ocular and nasal systems, digestive system, urogenital system, respiratory system, signs of discomfort, as well as the body weight (g) and body surface temperature (°C). A list of each potential deficit evaluated in this study is shown in the left hand column of the table. Each potential deficit could have a minimum score of 0 (no deficit present) and a maximum score of 1 (severe deficit). For each animal, the scores for each deficit were added and divided by the total number of deficits measured (e.g. 31) to yield af frailty index score of between 0 and 1. The average score (± SEM) for each potential deficit for all three age groups is also shown in the table.

Potential Deficit	Average Score for Each Potential Deficit (± SEM)		
	Young Adult	Older Adult	Aged
Alopecia (hair loss)	0	0.625 ± 0.239	0.500 ± 0.204
Loss of fur colour	0	0.250 ± 0.144	0.750 ± 0.144
Dermatitis	0	0	0.125 ± 0.125
Loss of whiskers	0	0	0.625 ± 0.125
Coat condition	0	0	0.125 ± 0.125
Tumours	0	0	0
Distended abdomen	0	0.125 ± 0.125	0.500 ± 0.204
Kyphosis/hunched posture	0	0	0.125 ± 0.125
Tail stiffening	0	0.500 ± 0	0.750 ± 0.144
Gait	0	0	0.375 ± 0.125
Tremor	0	0	0.375 ± 0.125
Forelimb grip strength	0.188 ± 0.091	0.125 ± 0.125	0.750 ± 0.144
Body condition score	0	0.250 ± 0.144	0.375 ± 0.239
Head tilt	0	0	0
Hearing loss	0.188 ± 0.132	0.375 ± 0.239	1.000 ± 0
Cataracts	0	0	0
Discharge/swollen/ squinting	0	0	0.125 ± 0.125
Microphthalmia	0	0	0
Corneal opacity	0	0	0
Vision loss	0	0	0.250 ± 0.144
Menace reflex	0.125 ± 0.082	0.125 ± 0.125	0.500 ± 0.204
Nasal discharge	0	0	0
Malocclusions	0	0	0.125 ± 0.125
Rectal prolapse	0	0	0.750 ± 0.144
Penile/Uterine prolapse	0	0	0
Diarrhea	0	0	0
Breathing rate/depth	0	0	0.500 ± 0.289
Mouse Grimace Scale	0	0	0
Piloerection	0	0	0
Body surface temperature	0.094 ± 0.046	0	0.062 ± 0.062
Body weight	0.031 ± 0.031	0.875 ± 0.125	0.625 ± 0.239
