# Supplementary material for: Microbial shifts in the aging mouse gut
Source: Microbiome. 2014 Dec 5;2:50. doi: 10.1186/s40168-014-0050-9 (PMC4269096; doi:10.1186/s40168-014-0050-9)
Supplement: Additional file 3: — PCA plot of relative OTU abundance. PCA plot based only on the relative OTU abundances in each sample, generated using the R function ‘prcomp’ shows separation of samples by age groups. [file 40168_2014_50_MOESM3_ESM.pdf]

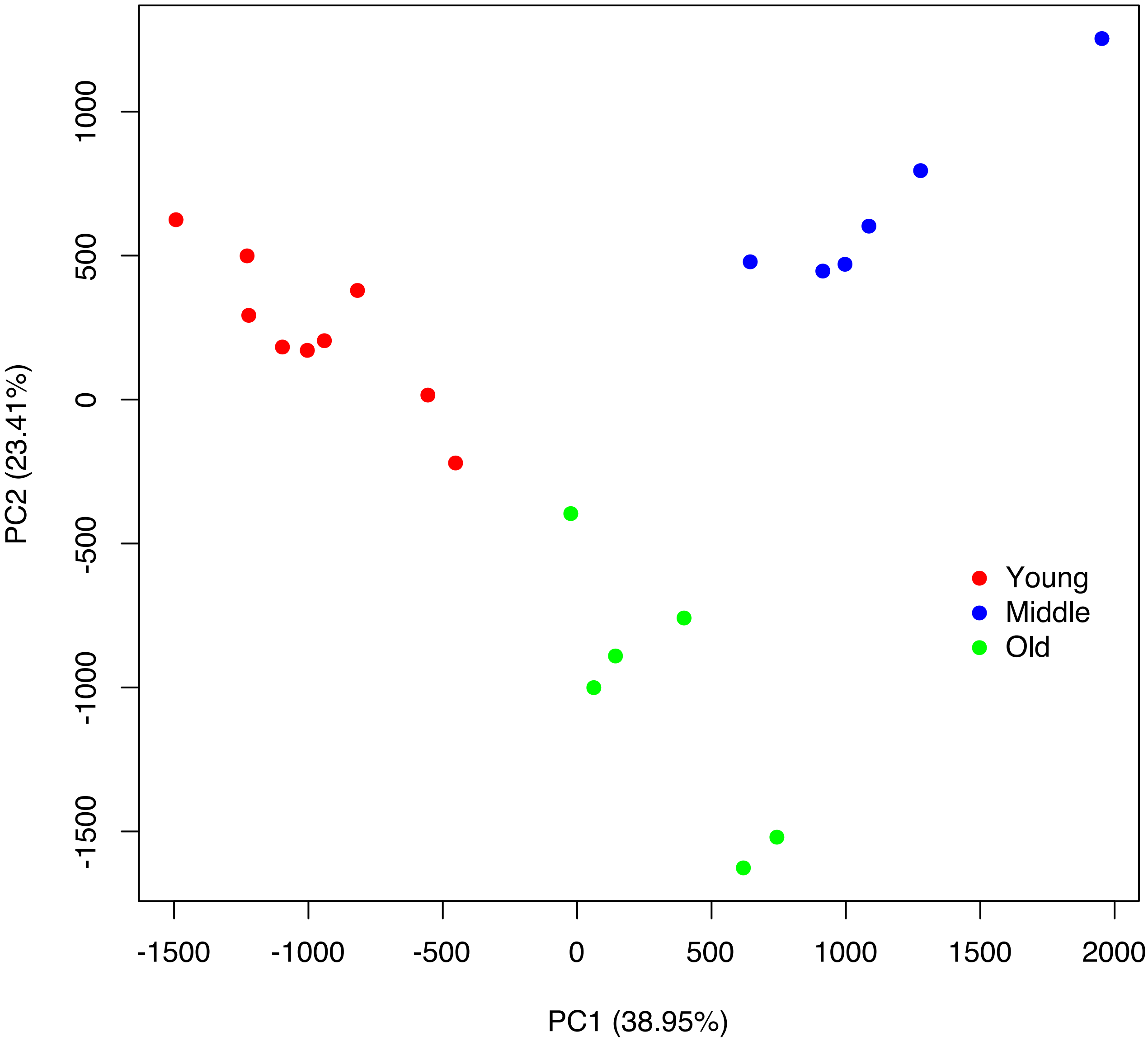

Additional File 3: PCA plot based only on the relative OTU abundances in each sample generated using the R function 'prcomp' show separation of samples by age groups.
