# Supplementary material for: Microbial shifts in the aging mouse gut
Source: Microbiome. 2014 Dec 5;2:50. doi: 10.1186/s40168-014-0050-9 (PMC4269096; doi:10.1186/s40168-014-0050-9)
Supplement: Additional file 4: — Pplacer edge PCA plot. Taxonomic separation of samples from protein-coding metagenome markers using the PhyloSift and Pplacer packages is shown using an edge PCA plot (A) with taxa contributing to the signal shown for PC2 (B) and PC3 (C). Taxa contributing to the positive direction of the PC are shown in orange while those contributing in the negative direction of the PC are shown in green. Branches that did not contribute to the PC were pruned from the tree. Taxon branches are collapsed to family level where the overrepresentation inferred by Pplacer is not restricted to a single species. Numbers within brackets are the count of taxa within that family. [file 40168_2014_50_MOESM4_ESM.pdf]

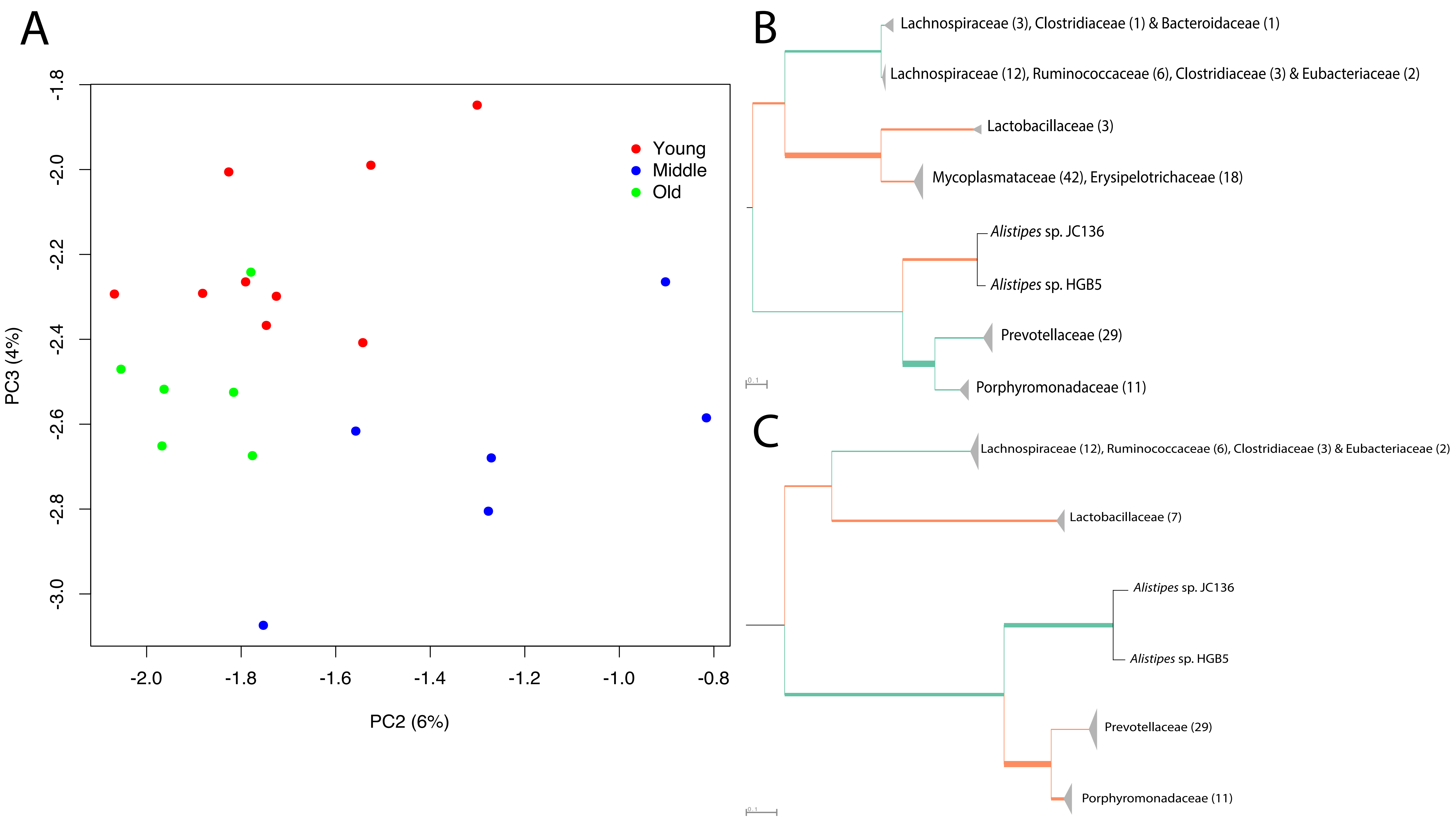

Additional File 4: Taxonomic separation of samples from protein-coding metagenome markers using the Phylosift and Pplacer packages is visualized using an edge PCA plot A) with taxa contributing to the signal shown for PC2 (B) and PC3 (C). Taxa contributing to the positive direction of the PC are shown in orange while those contributing in the negative direction of the PC are shown in green. Branches that did not contribute to the PC were pruned from the tree. Taxon branches are collapsed to family level where the significant over-representation is not to a specific species. Numbers within brackets are the count of taxa within that family.
