# Supplementary material for: Microbial shifts in the aging mouse gut
Source: Microbiome. 2014 Dec 5;2:50. doi: 10.1186/s40168-014-0050-9 (PMC4269096; doi:10.1186/s40168-014-0050-9)
Supplement: Additional file 5: — Variation within samples taken from same and different mice. Comparison of beta-diversity measurements between samples taken from either the same mouse or a different mouse for each age group (young, middle, old) using weighted UniFrac (A) or unweighted UniFrac (B). [file 40168_2014_50_MOESM5_ESM.pdf]

A

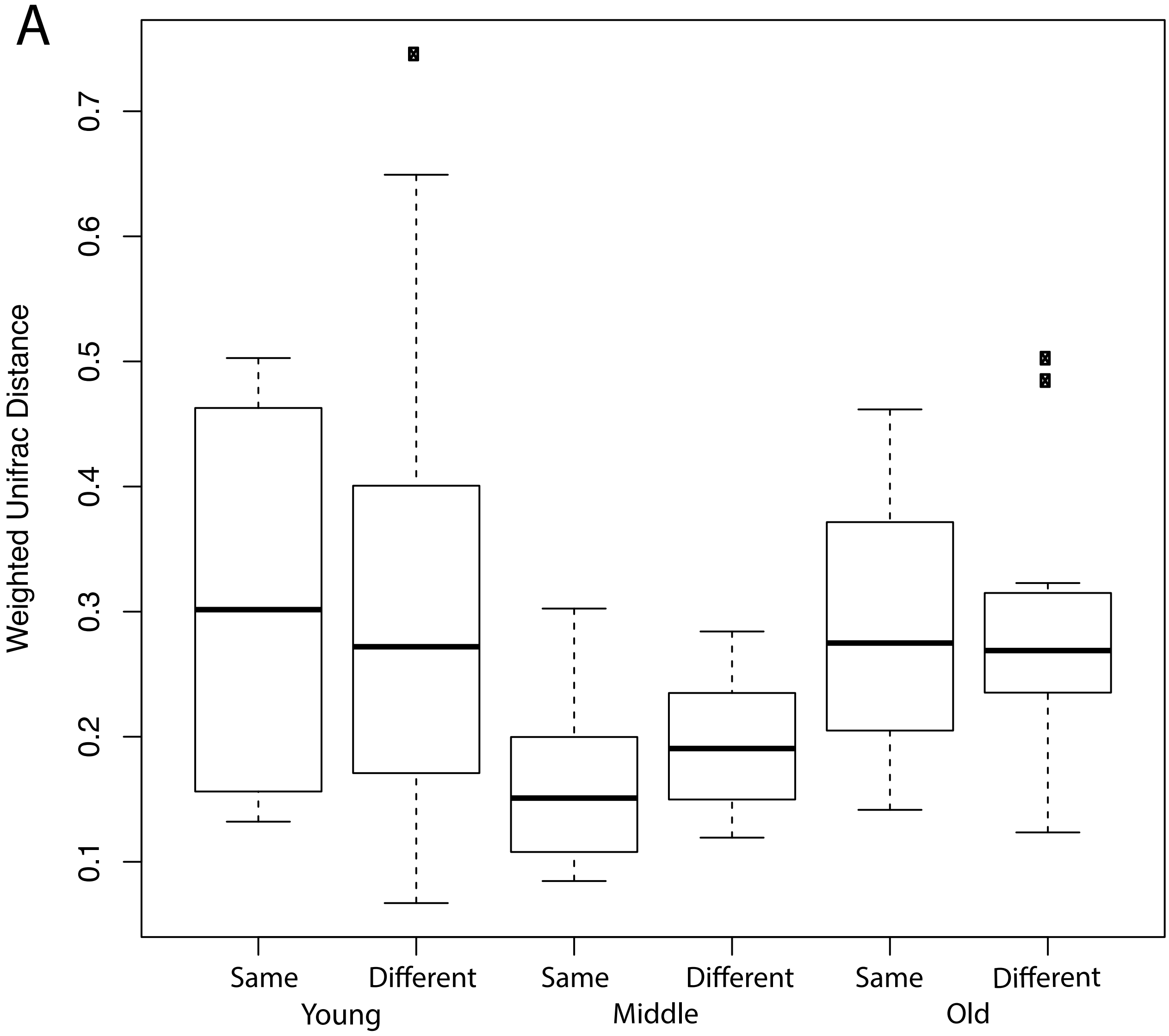

B

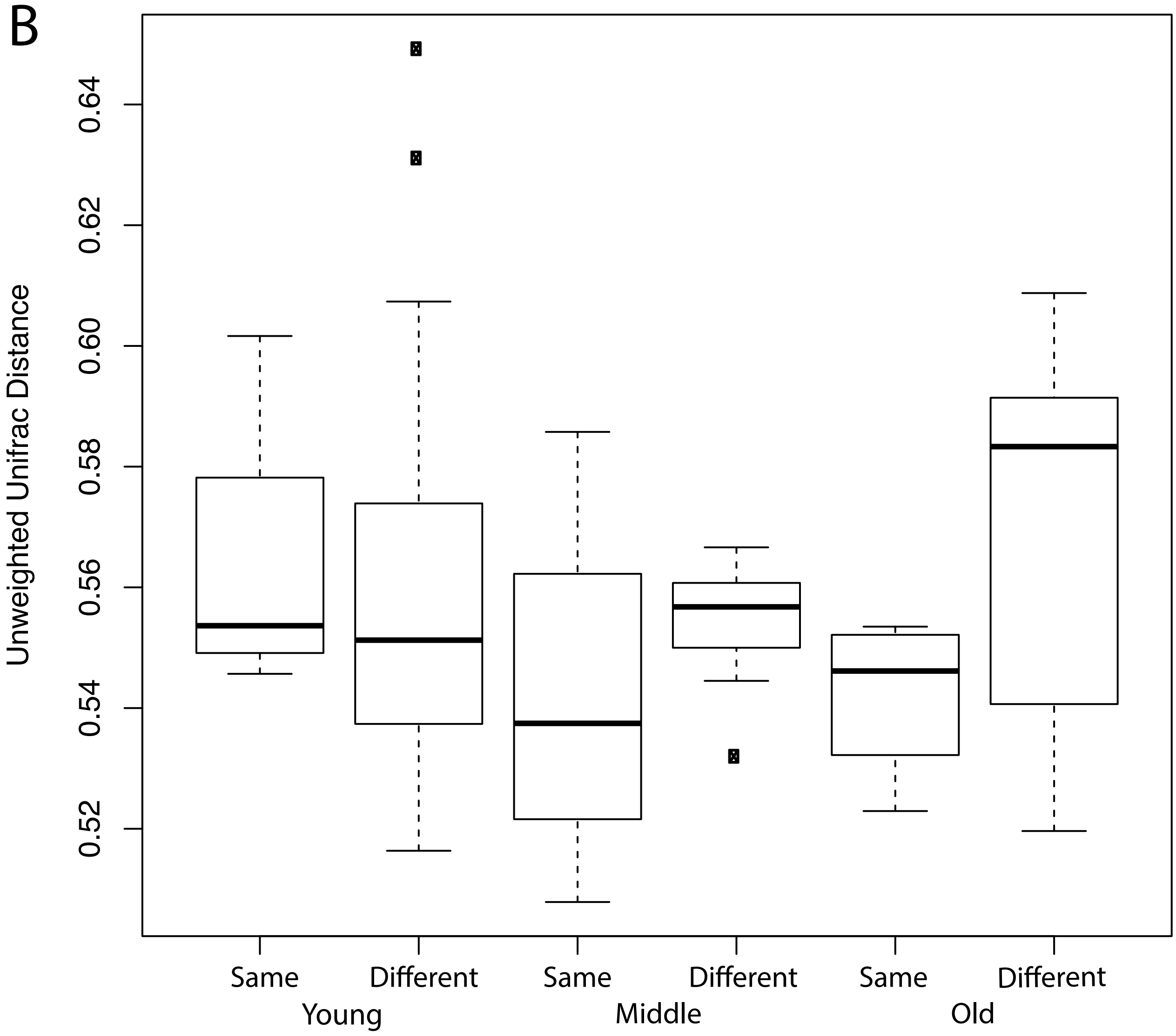

Additional File 5: Comparison of beta-diversity measurements between samples taken from either the same mouse or a different mouse for each age group (Young, Middle, Old) using weighted UniFrac (A) or unweighted UniFrac (B).
