# Supplementary material for: Microbial shifts in the aging mouse gut
Source: Microbiome. 2014 Dec 5;2:50. doi: 10.1186/s40168-014-0050-9 (PMC4269096; doi:10.1186/s40168-014-0050-9)
Supplement: Additional file 7: — Taxonomic composition of samples. Taxonomic composition identified using 16S sequences for all 21 samples ordered by increasing age and frailty from left to right at the phylum (A), family (B), and genus (C) levels. [file 40168_2014_50_MOESM7_ESM.pdf]

A)

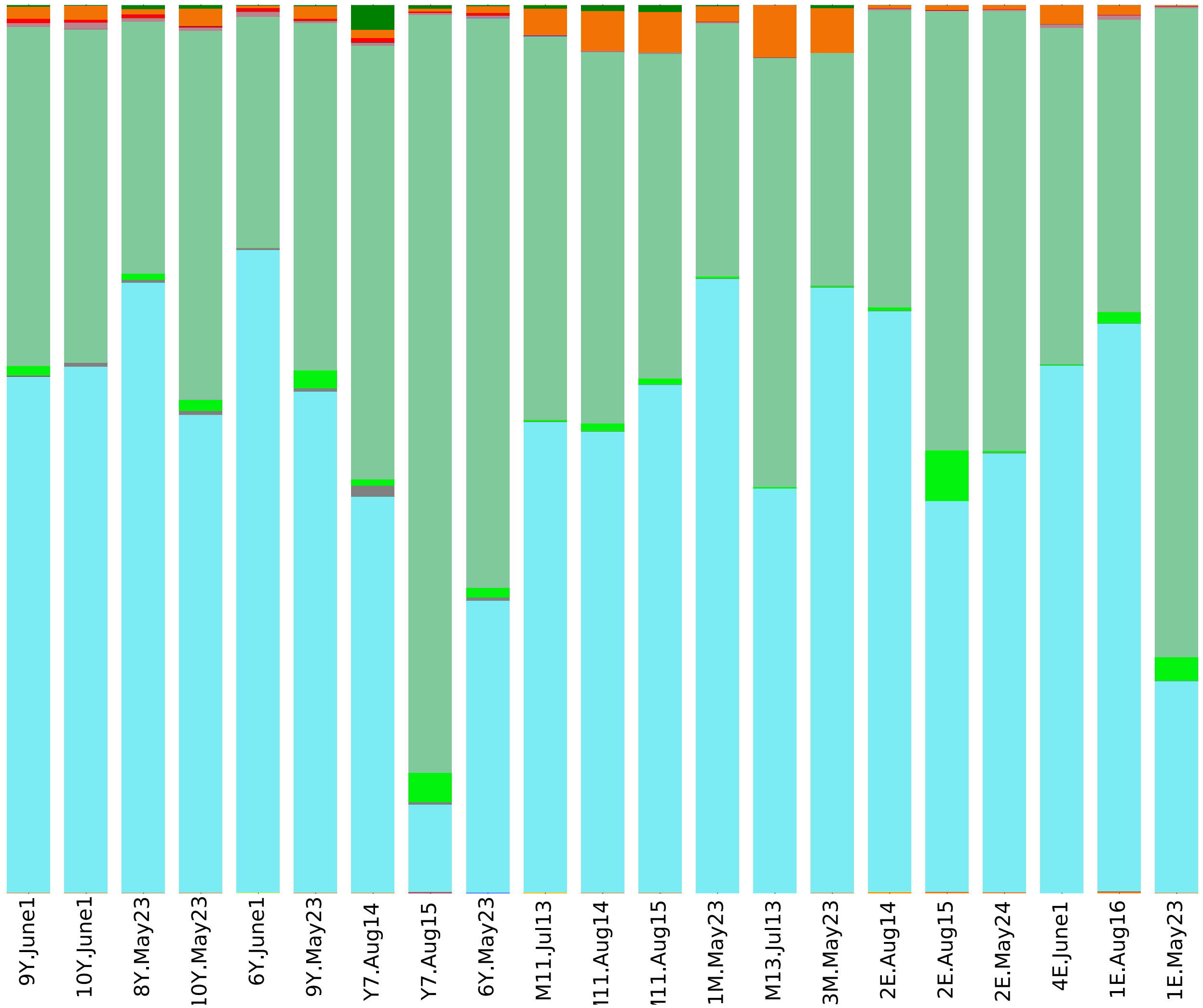

Additional File 7: Taxonomic composition identified using 16S sequences for all 21 samples ordered by increasing age and frailty from left to right at the phylum (A), family (B), and genus (C) levels.

k__Bacteria; p__
k__Bacteria; p__Acidobacteria
k__Bacteria; p__Actinobacteria
k__Bacteria; p__Armatimonadetes
k__Bacteria; p__BHI80-139
k__Bacteria; p__BRC1
k__Bacteria; p__Bacteroidetes
k__Bacteria; p__Caldithrix
k__Bacteria; p__Chlorobi
k__Bacteria; p__Chloroflexi
k__Bacteria; p__Cyanobacteria
k__Bacteria; p__Deferribacteres
k__Bacteria; p__Elusimicrobia
k__Bacteria; p__FBP
k__Bacteria; p__Fibrobacteres
k__Bacteria; p__Firmicutes
k__Bacteria; p__Fusobacteria
k__Bacteria; p__GN04
k__Bacteria; p__GOUTA4
k__Bacteria; p__Gemmatimonadetes
k__Bacteria; p__Hyd24-12
k__Bacteria; p__Nitrospirae
k__Bacteria; p__OP8
k__Bacteria; p__Planctomycetes
k__Bacteria; p__Proteobacteria
k__Bacteria; p__SAR406
k__Bacteria; p__Spirochaetes
k__Bacteria; p__Synergistetes
k__Bacteria; p__TM6
k__Bacteria; p__TM7
k__Bacteria; p__TPD-58
k__Bacteria; p__Tenericutes
k__Bacteria; p__Verrucomicrobia
k__Bacteria; p__WPS-2
k__Bacteria; p__WS1
k__Bacteria; p__WS3
k__Bacteria; p__ZB3
k__Bacteria; p__[Thermi]

B)

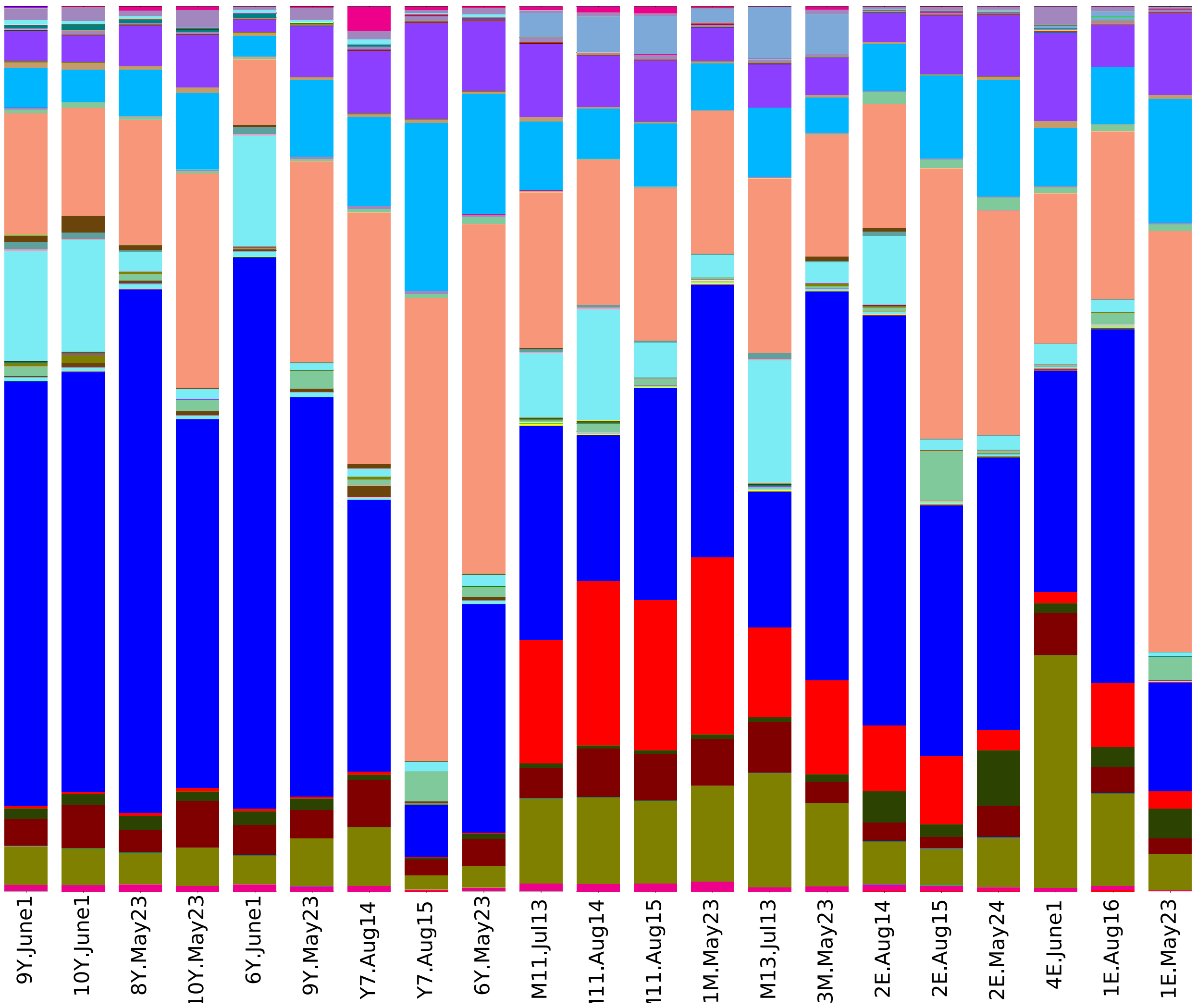

9Y.June1
10Y.June1
8Y.May23
10Y.May23
6Y.June1
9Y.May23
Y7.Aug14
Y7.Aug15
6Y.May23
M11.Jul13
I11.Aug14
I11.Aug15
1M.May23
M13.Jul13
3M.May23
2E.Aug14
2E.Aug15
2E.May24
4E.June1
1E.Aug16
1E.May23

k__Bacteria; p__; c__; o__; f__
k__Bacteria; p__Acidobacteria; c__Acidobacteria-6; o__iii1-15; f__
k__Bacteria; p__Acidobacteria; c__Acidobacteria-6; o__iii1-15; f__RB40
k__Bacteria; p__Acidobacteria; c__Acidobacteria-6; o__iii1-15; f__mb2424
k__Bacteria; p__Acidobacteria; c__Acidobacteriia; o__Acidobacteriales; f__Acidobacteriaceae
k__Bacteria; p__Acidobacteria; c__Acidobacteriia; o__Acidobacteriales; f__Koribacteraceae
k__Bacteria; p__Acidobacteria; c__BPC102; o__B110; f__
k__Bacteria; p__Acidobacteria; c__Holophagae; o__Holophagales; f__Holophagaceae
k__Bacteria; p__Acidobacteria; c__OS-K; o__; f__
k__Bacteria; p__Acidobacteria; c__RB25; o__; f__
k__Bacteria; p__Acidobacteria; c__Solibacteres; o__Solibacterales; f__
k__Bacteria; p__Acidobacteria; c__Sva0725; o__Sva0725; f__
k__Bacteria; p__Acidobacteria; c__[Chloracidobacteria]; o__PK29; f__
k__Bacteria; p__Acidobacteria; c__[Chloracidobacteria]; o__RB41; f__
k__Bacteria; p__Acidobacteria; c__[Chloracidobacteria]; o__RB41; f__Ellin6075
k__Bacteria; p__Actinobacteria; c__Acidimicrobiia; o__Acidimicrobiales; f__
k__Bacteria; p__Actinobacteria; c__Acidimicrobiia; o__Acidimicrobiales; f__koll13
k__Bacteria; p__Actinobacteria; c__Actinobacteria; o__Actinomycetales; f__Actinomycetaceae
k__Bacteria; p__Actinobacteria; c__Actinobacteria; o__Actinomycetales; f__Brevibacteriaceae
k__Bacteria; p__Actinobacteria; c__Actinobacteria; o__Actinomycetales; f__Cellulomonadaceae
k__Bacteria; p__Actinobacteria; c__Actinobacteria; o__Actinomycetales; f__Corynebacteriaceae
k__Bacteria; p__Actinobacteria; c__Actinobacteria; o__Actinomycetales; f__Dermabacteraceae
k__Bacteria; p__Actinobacteria; c__Actinobacteria; o__Actinomycetales; f__Microbacteriaceae
k__Bacteria; p__Actinobacteria; c__Actinobacteria; o__Actinomycetales; f__Micrococcaceae
k__Bacteria; p__Actinobacteria; c__Actinobacteria; o__Actinomycetales; f__Mycobacteriaceae
k__Bacteria; p__Actinobacteria; c__Actinobacteria; o__Actinomycetales; f__Nocardiaceae
k__Bacteria; p__Actinobacteria; c__Actinobacteria; o__Actinomycetales; f__Pseudonocardiaceae
k__Bacteria; p__Actinobacteria; c__Actinobacteria; o__Actinomycetales; f__Yaniellaceae
k__Bacteria; p__Actinobacteria; c__Actinobacteria; o__Bifidobacteriales; f__Bifidobacteriaceae
k__Bacteria; p__Actinobacteria; c__Coriobacteriia; o__Coriobacteriales; f__Coriobacteriaceae
k__Bacteria; p__Actinobacteria; c__MB-A2-108; o__; f__
k__Bacteria; p__Actinobacteria; c__MB-A2-108; o__0319-7L14; f__
k__Bacteria; p__Actinobacteria; c__Nitriliruptoria; o__Nitriliruptorales; f__Nitriliruptoraceae
k__Bacteria; p__Actinobacteria; c__OPB41; o__; f__
k__Bacteria; p__Actinobacteria; c__Rubrobacteria; o__Rubrobacterales; f__Rubrobacteraceae
k__Bacteria; p__Actinobacteria; c__Thermoleophilia; o__Gaiellales; f__
k__Bacteria; p__Actinobacteria; c__Thermoleophilia; o__Gaiellales; f__Gaiellaceae
k__Bacteria; p__Actinobacteria; c__Thermoleophilia; o__Solirubrobacterales; f__
k__Bacteria; p__Actinobacteria; c__Thermoleophilia; o__Solirubrobacterales; f__Patulibacteraceae
k__Bacteria; p__Armatimonadetes; c__0319-6E2; o__; f__
k__Bacteria; p__Armatimonadetes; c__Chthonomonadetes; o__SJA-22; f__
k__Bacteria; p__Armatimonadetes; c__SJA-176; o__RB046; f__
k__Bacteria; p__Armatimonadetes; c__[Fimbriimonadia]; o__[Fimbriimonadales]; f__[Fimbriimonadaceae]
k__Bacteria; p__BHI80-139; c__; o__; f__
k__Bacteria; p__BRC1; c__NPL-UPA2; o__; f__
k__Bacteria; p__BRC1; c__PRR-11; o__; f__
k__Bacteria; p__Bacteroidetes; c__; o__; f__
k__Bacteria; p__Bacteroidetes; c__BME43; o__; f__
k__Bacteria; p__Bacteroidetes; c__Bacteroidia; o__Bacteroidales; f__
k__Bacteria; p__Bacteroidetes; c__Bacteroidia; o__Bacteroidales; f__BA008
k__Bacteria; p__Bacteroidetes; c__Bacteroidia; o__Bacteroidales; f__BS11
k__Bacteria; p__Bacteroidetes; c__Bacteroidia; o__Bacteroidales; f__Bacteroidaceae
k__Bacteria; p__Bacteroidetes; c__Bacteroidia; o__Bacteroidales; f__GZKB119
k__Bacteria; p__Bacteroidetes; c__Bacteroidia; o__Bacteroidales; f__ML635J-40
k__Bacteria; p__Bacteroidetes; c__Bacteroidia; o__Bacteroidales; f__Marinilabiaceae
k__Bacteria; p__Bacteroidetes; c__Bacteroidia; o__Bacteroidales; f__Porphyromonadaceae
k__Bacteria; p__Bacteroidetes; c__Bacteroidia; o__Bacteroidales; f__Prevotellaceae
k__Bacteria; p__Bacteroidetes; c__Bacteroidia; o__Bacteroidales; f__RF16
k__Bacteria; p__Bacteroidetes; c__Bacteroidia; o__Bacteroidales; f__Rikenellaceae
k__Bacteria; p__Bacteroidetes; c__Bacteroidia; o__Bacteroidales; f__S24-7
k__Bacteria; p__Bacteroidetes; c__Bacteroidia; o__Bacteroidales; f__SB-1
k__Bacteria; p__Bacteroidetes; c__Bacteroidia; o__Bacteroidales; f__VC21_Bac22
k__Bacteria; p__Bacteroidetes; c__Bacteroidia; o__Bacteroidales; f__[Barnesiellaceae]
k__Bacteria; p__Bacteroidetes; c__Bacteroidia; o__Bacteroidales; f__[Odoribacteraceae]
k__Bacteria; p__Bacteroidetes; c__Bacteroidia; o__Bacteroidales; f__[Paraprevotellaceae]
k__Bacteria; p__Bacteroidetes; c__Bacteroidia; o__Bacteroidales; f__p-2534-18B5
k__Bacteria; p__Bacteroidetes; c__Cytophagia; o__Cytophagales; f__Cyclobacteriaceae
k__Bacteria; p__Bacteroidetes; c__Cytophagia; o__Cytophagales; f__Cytophagaceae
k__Bacteria; p__Bacteroidetes; c__Cytophagia; o__Cytophagales; f__Flammeovirgaceae
k__Bacteria; p__Bacteroidetes; c__Flavobacteriia; o__; f__
k__Bacteria; p__Bacteroidetes; c__Flavobacteriia; o__Flavobacteriales; f__
k__Bacteria; p__Bacteroidetes; c__Flavobacteriia; o__Flavobacteriales; f__Cryomorphaceae
k__Bacteria; p__Bacteroidetes; c__Flavobacteriia; o__Flavobacteriales; f__Flavobacteriaceae
k__Bacteria; p__Bacteroidetes; c__Flavobacteriia; o__Flavobacteriales; f__NS9
k__Bacteria; p__Bacteroidetes; c__Flavobacteriia; o__Flavobacteriales; f__[Weeksellaceae]
k__Bacteria; p__Bacteroidetes; c__Sphingobacteriia; o__Sphingobacteriales; f__
k__Bacteria; p__Bacteroidetes; c__Sphingobacteriia; o__Sphingobacteriales; f__Sphingobacteriaceae
k__Bacteria; p__Bacteroidetes; c__[Rhodothermi]; o__[Rhodothermales]; f__Rhodothermaceae
k__Bacteria; p__Bacteroidetes; c__[Saprospirae]; o__[Saprospirales]; f__
k__Bacteria; p__Bacteroidetes; c__[Saprospirae]; o__[Saprospirales]; f__Chitinophagaceae
k__Bacteria; p__Bacteroidetes; c__[Saprospirae]; o__[Saprospirales]; f__Saprospiraceae
k__Bacteria; p__Caldithrix; c__Caldithrixae; o__Caldithrixales; f__Caldithrixaceae
k__Bacteria; p__Chlorobi; c__; o__; f__
k__Bacteria; p__Chlorobi; c__BSV26; o__A89; f__
k__Bacteria; p__Chlorobi; c__BSV26; o__C20; f__
k__Bacteria; p__Chlorobi; c__BSV26; o__PK329; f__
k__Bacteria; p__Chlorobi; c__Ignavibacteria; o__Ignavibacteriales; f__
k__Bacteria; p__Chlorobi; c__Ignavibacteria; o__Ignavibacteriales; f__Ignavibacteriaceae
k__Bacteria; p__Chlorobi; c__Ignavibacteria; o__Ignavibacteriales; f__[Melioribacteraceae]
k__Bacteria; p__Chlorobi; c__Ignavibacteria; o__Ignavibacteriales; f__lheB3-7
k__Bacteria; p__Chlorobi; c__OPB56; o__; f__
k__Bacteria; p__Chloroflexi; c__Anaerolineae; o__CFB-26; f__
k__Bacteria; p__Chloroflexi; c__Anaerolineae; o__Caldilineales; f__Caldilineaceae
k__Bacteria; p__Chloroflexi; c__Dehalococcoidetes; o__Dehalococcoidales; f__Dehalococcoidaceae
k__Bacteria; p__Chloroflexi; c__S085; o__; f__
k__Bacteria; p__Cyanobacteria; c__4C0d-2; o__SM1D11; f__
k__Bacteria; p__Cyanobacteria; c__4C0d-2; o__YS2; f__
k__Bacteria; p__Cyanobacteria; c__Chloroplast; o__Streptophyta; f__
k__Bacteria; p__Cyanobacteria; c__Gloeobacterophycideae; o__Gloeobacterales; f__Gloeobacteraceae
k__Bacteria; p__Cyanobacteria; c__Nostocophycideae; o__Stigonematales; f__Rivulariaceae
k__Bacteria; p__Cyanobacteria; c__Synechococcophycideae; o__Pseudanabaenales; f__Pseudanabaenaceae
k__Bacteria; p__Deferribacteres; c__Deferribacteres; o__Deferribacterales; f__
k__Bacteria; p__Deferribacteres; c__Deferribacteres; o__Deferribacterales; f__Deferribacteraceae
k__Bacteria; p__Elusimicrobia; c__Elusimicrobia; o__Elusimicrobiales; f__
k__Bacteria; p__FBP; c__; o__; f__
k__Bacteria; p__Fibrobacteres; c__B5-096; o__; f__
k__Bacteria; p__Firmicutes; c__AHT28; o__; f__
k__Bacteria; p__Firmicutes; c__Bacilli; o__Bacillales; f__
k__Bacteria; p__Firmicutes; c__Bacilli; o__Bacillales; f__Alicyclobacillaceae
k__Bacteria; p__Firmicutes; c__Bacilli; o__Bacillales; f__Bacillaceae
k__Bacteria; p__Firmicutes; c__Bacilli; o__Bacillales; f__Paenibacillaceae
k__Bacteria; p__Firmicutes; c__Bacilli; o__Bacillales; f__Planococcaceae
k__Bacteria; p__Firmicutes; c__Bacilli; o__Bacillales; f__Staphylococcaceae
k__Bacteria; p__Firmicutes; c__Bacilli; o__Bacillales; f__Thermoactinomycetaceae
k__Bacteria; p__Firmicutes; c__Bacilli; o__Bacillales; f__[Exiguobacteraceae]
k__Bacteria; p__Firmicutes; c__Bacilli; o__Gemellales; f__
k__Bacteria; p__Firmicutes; c__Bacilli; o__Gemellales; f__Gemellaceae
k__Bacteria; p__Firmicutes; c__Bacilli; o__Haloplasmatales; f__Haloplasmataceae
k__Bacteria; p__Firmicutes; c__Bacilli; o__Lactobacillales; f__
k__Bacteria; p__Firmicutes; c__Bacilli; o__Lactobacillales; f__Aerococcaceae
k__Bacteria; p__Firmicutes; c__Bacilli; o__Lactobacillales; f__Carnobacteriaceae
k__Bacteria; p__Firmicutes; c__Bacilli; o__Lactobacillales; f__Enterococcaceae
k__Bacteria; p__Firmicutes; c__Bacilli; o__Lactobacillales; f__Lactobacillaceae
k__Bacteria; p__Firmicutes; c__Bacilli; o__Lactobacillales; f__Leuconostocaceae
k__Bacteria; p__Firmicutes; c__Bacilli; o__Lactobacillales; f__Streptococcaceae
k__Bacteria; p__Firmicutes; c__Bacilli; o__Turicibacterales; f__Turicibacteraceae
k__Bacteria; p__Firmicutes; c__Clostridia; o__; f__
k__Bacteria; p__Firmicutes; c__Clostridia; o__BSA2B-08; f__
k__Bacteria; p__Firmicutes; c__Clostridia; o__Clostridiales; f__
k__Bacteria; p__Firmicutes; c__Clostridia; o__Clostridiales; f__Caldicoprobacteraceae
k__Bacteria; p__Firmicutes; c__Clostridia; o__Clostridiales; f__Christensenellaceae
k__Bacteria; p__Firmicutes; c__Clostridia; o__Clostridiales; f__Clostridiaceae
k__Bacteria; p__Firmicutes; c__Clostridia; o__Clostridiales; f__Dehalobacteriaceae
k__Bacteria; p__Firmicutes; c__Clostridia; o__Clostridiales; f__Eubacteriaceae
k__Bacteria; p__Firmicutes; c__Clostridia; o__Clostridiales; f__Gracilibacteraceae
k__Bacteria; p__Firmicutes; c__Clostridia; o__Clostridiales; f__Heliobacteriaceae
k__Bacteria; p__Firmicutes; c__Clostridia; o__Clostridiales; f__Lachnospiraceae
k__Bacteria; p__Firmicutes; c__Clostridia; o__Clostridiales; f__Peptococcaceae
k__Bacteria; p__Firmicutes; c__Clostridia; o__Clostridiales; f__Peptostreptococcaceae
k__Bacteria; p__Firmicutes; c__Clostridia; o__Clostridiales; f__Ruminococcaceae
k__Bacteria; p__Firmicutes; c__Clostridia; o__Clostridiales; f__SBYG_4172
k__Bacteria; p__Firmicutes; c__Clostridia; o__Clostridiales; f__Syntrophomonadaceae
k__Bacteria; p__Firmicutes; c__Clostridia; o__Clostridiales; f__Veillonellaceae
k__Bacteria; p__Firmicutes; c__Clostridia; o__Clostridiales; f__[Acidaminobacteraceae]
k__Bacteria; p__Firmicutes; c__Clostridia; o__Clostridiales; f__[Mogibacteriaceae]
k__Bacteria; p__Firmicutes; c__Clostridia; o__Clostridiales; f__[Tissierellaceae]
k__Bacteria; p__Firmicutes; c__Clostridia; o__Halanaerobiales; f__
k__Bacteria; p__Firmicutes; c__Clostridia; o__Halanaerobiales; f__Halanaerobiaceae
k__Bacteria; p__Firmicutes; c__Clostridia; o__Halanaerobiales; f__Halobacteroidaceae
k__Bacteria; p__Firmicutes; c__Clostridia; o__MBA08; f__
k__Bacteria; p__Firmicutes; c__Clostridia; o__Natranaerobiales; f__
k__Bacteria; p__Firmicutes; c__Clostridia; o__Natranaerobiales; f__Anaerobrancaceae
k__Bacteria; p__Firmicutes; c__Clostridia; o__Natranaerobiales; f__Contubernalisaceae
k__Bacteria; p__Firmicutes; c__Clostridia; o__Natranaerobiales; f__ML1228J-1
k__Bacteria; p__Firmicutes; c__Clostridia; o__OPB54; f__
k__Bacteria; p__Firmicutes; c__Clostridia; o__SHA-98; f__
k__Bacteria; p__Firmicutes; c__Clostridia; o__SHA-98; f__D2
k__Bacteria; p__Firmicutes; c__Clostridia; o__Thermoanaerobacterales; f__
k__Bacteria; p__Firmicutes; c__Clostridia; o__Thermoanaerobacterales; f__Caldicellulosiruptoraceae
k__Bacteria; p__Firmicutes; c__Clostridia; o__Thermoanaerobacterales; f__Thermoanaerobacteraceae
k__Bacteria; p__Firmicutes; c__Clostridia; o__Thermoanaerobacterales; f__Thermodesulfobiaceae
k__Bacteria; p__Firmicutes; c__Erysipelotrichi; o__Erysipelotrichales; f__Erysipelotrichaceae
k__Bacteria; p__Fusobacteria; c__Fusobacteriia; o__Fusobacteriales; f__Fusobacteriaceae
k__Bacteria; p__GN04; c__MSB-5A5; o__; f__
k__Bacteria; p__GOUTA4; c__; o__; f__
k__Bacteria; p__Gemmatimonadetes; c__; o__; f__
k__Bacteria; p__Gemmatimonadetes; c__Gemm-1; o__; f__
k__Bacteria; p__Gemmatimonadetes; c__Gemm-3; o__; f__
k__Bacteria; p__Gemmatimonadetes; c__JL-ETNP-Z39; o__; f__
k__Bacteria; p__Hyd24-12; c__WM88; o__; f__
k__Bacteria; p__Nitrospirae; c__Nitrospira; o__Nitrospirales; f__0319-6A21
k__Bacteria; p__Nitrospirae; c__Nitrospira; o__Nitrospirales; f__Nitrospiraceae
k__Bacteria; p__OP8; c__; o__; f__
k__Bacteria; p__OP8; c__OP8_1; o__; f__
k__Bacteria; p__Planctomycetes; c__Phycisphaerae; o__Phycisphaerales; f__
k__Bacteria; p__Planctomycetes; c__Planctomycetia; o__Gemmatales; f__Isosphaeraceae
k__Bacteria; p__Planctomycetes; c__Planctomycetia; o__Pirellulales; f__Pirellulaceae
k__Bacteria; p__Proteobacteria; c__; o__; f__
k__Bacteria; p__Proteobacteria; c__Alphaproteobacteria; o__; f__
k__Bacteria; p__Proteobacteria; c__Alphaproteobacteria; o__Caulobacterales; f__
k__Bacteria; p__Proteobacteria; c__Alphaproteobacteria; o__Caulobacterales; f__Caulobacteraceae
k__Bacteria; p__Proteobacteria; c__Alphaproteobacteria; o__Kiloniellales; f__Kiloniellaceae
k__Bacteria; p__Proteobacteria; c__Alphaproteobacteria; o__Rhizobiales; f__
k__Bacteria; p__Proteobacteria; c__Alphaproteobacteria; o__Rhizobiales; f__Aurantimonadaceae
k__Bacteria; p__Proteobacteria; c__Alphaproteobacteria; o__Rhizobiales; f__Hyphomicrobiaceae
k__Bacteria; p__Proteobacteria; c__Alphaproteobacteria; o__Rhizobiales; f__Methylocystaceae
k__Bacteria; p__Proteobacteria; c__Alphaproteobacteria; o__Rhizobiales; f__Rhizobiaceae
k__Bacteria; p__Proteobacteria; c__Alphaproteobacteria; o__Rhodobacterales; f__Rhodobacteraceae
k__Bacteria; p__Proteobacteria; c__Alphaproteobacteria; o__Rhodospirillales; f__
k__Bacteria; p__Proteobacteria; c__Alphaproteobacteria; o__Rhodospirillales; f__Acetobacteraceae
k__Bacteria; p__Proteobacteria; c__Alphaproteobacteria; o__Rhodospirillales; f__Rhodospirillaceae
k__Bacteria; p__Proteobacteria; c__Alphaproteobacteria; o__Rickettsiales; f__
k__Bacteria; p__Proteobacteria; c__Alphaproteobacteria; o__Rickettsiales; f__Pelagibacteraceae
k__Bacteria; p__Proteobacteria; c__Alphaproteobacteria; o__Rickettsiales; f__mitochondria
k__Bacteria; p__Proteobacteria; c__Alphaproteobacteria; o__Sphingomonadales; f__Erythrobacteraceae
k__Bacteria; p__Proteobacteria; c__Alphaproteobacteria; o__Sphingomonadales; f__Sphingomonadaceae
k__Bacteria; p__Proteobacteria; c__Betaproteobacteria; o__; f__
k__Bacteria; p__Proteobacteria; c__Betaproteobacteria; o__A21b; f__EB1003
k__Bacteria; p__Proteobacteria; c__Betaproteobacteria; o__Burkholderiales; f__
k__Bacteria; p__Proteobacteria; c__Betaproteobacteria; o__Burkholderiales; f__Alcaligenaceae
k__Bacteria; p__Proteobacteria; c__Betaproteobacteria; o__Burkholderiales; f__Burkholderiaceae
k__Bacteria; p__Proteobacteria; c__Betaproteobacteria; o__Burkholderiales; f__Comamonadaceae
k__Bacteria; p__Proteobacteria; c__Betaproteobacteria; o__Burkholderiales; f__Oxalobacteraceae
k__Bacteria; p__Proteobacteria; c__Betaproteobacteria; o__Gallionellales; f__Gallionellaceae
k__Bacteria; p__Proteobacteria; c__Betaproteobacteria; o__MND1; f__
k__Bacteria; p__Proteobacteria; c__Betaproteobacteria; o__MWH-UniP1; f__
k__Bacteria; p__Proteobacteria; c__Betaproteobacteria; o__Methylophilales; f__Methylophilaceae
k__Bacteria; p__Proteobacteria; c__Betaproteobacteria; o__Neisseriales; f__Neisseriaceae
k__Bacteria; p__Proteobacteria; c__Betaproteobacteria; o__Procabacteriales; f__Procabacteriaceae
k__Bacteria; p__Proteobacteria; c__Betaproteobacteria; o__Rhodocyclales; f__Rhodocyclaceae
k__Bacteria; p__Proteobacteria; c__Betaproteobacteria; o__SC-I-84; f__
k__Bacteria; p__Proteobacteria; c__Betaproteobacteria; o__Thiobacterales; f__
k__Bacteria; p__Proteobacteria; c__Deltaproteobacteria; o__Bdellovibrionales; f__Bdellovibrionaceae
k__Bacteria; p__Proteobacteria; c__Deltaproteobacteria; o__Desulfarculales; f__Desulfarculaceae
k__Bacteria; p__Proteobacteria; c__Deltaproteobacteria; o__Desulfobacterales; f__Desulfobacteraceae
k__Bacteria; p__Proteobacteria; c__Deltaproteobacteria; o__Desulfobacterales; f__Desulfobulbaceae
k__Bacteria; p__Proteobacteria; c__Deltaproteobacteria; o__Desulfovibrionales; f__Desulfovibrionaceae
k__Bacteria; p__Proteobacteria; c__Deltaproteobacteria; o__Desulfuromonadales; f__Geobacteraceae
k__Bacteria; p__Proteobacteria; c__Deltaproteobacteria; o__IndB3-24; f__
k__Bacteria; p__Proteobacteria; c__Deltaproteobacteria; o__MIZ46; f__
k__Bacteria; p__Proteobacteria; c__Deltaproteobacteria; o__Myxococcales; f__
k__Bacteria; p__Proteobacteria; c__Deltaproteobacteria; o__PB19; f__
k__Bacteria; p__Proteobacteria; c__Deltaproteobacteria; o__Spirobacillales; f__
k__Bacteria; p__Proteobacteria; c__Deltaproteobacteria; o__Syntrophobacterales; f__
k__Bacteria; p__Proteobacteria; c__Deltaproteobacteria; o__Syntrophobacterales; f__Syntrophaceae
k__Bacteria; p__Proteobacteria; c__Deltaproteobacteria; o__Syntrophobacterales; f__Syntrophobacteraceae
k__Bacteria; p__Proteobacteria; c__Epsilonproteobacteria; o__Campylobacterales; f__Campylobacteraceae
k__Bacteria; p__Proteobacteria; c__Epsilonproteobacteria; o__Campylobacterales; f__Helicobacteraceae
k__Bacteria; p__Proteobacteria; c__Gammaproteobacteria; o__; f__
k__Bacteria; p__Proteobacteria; c__Gammaproteobacteria; o__34P16; f__
k__Bacteria; p__Proteobacteria; c__Gammaproteobacteria; o__Acidithiobacillales; f__Acidithiobacillaceae
k__Bacteria; p__Proteobacteria; c__Gammaproteobacteria; o__Aeromonadales; f__Succinivibrionaceae
k__Bacteria; p__Proteobacteria; c__Gammaproteobacteria; o__Alteromonadales; f__Alteromonadaceae
k__Bacteria; p__Proteobacteria; c__Gammaproteobacteria; o__Alteromonadales; f__Colwelliaceae
k__Bacteria; p__Proteobacteria; c__Gammaproteobacteria; o__Alteromonadales; f__Shewanellaceae
k__Bacteria; p__Proteobacteria; c__Gammaproteobacteria; o__Cardiobacteriales; f__
k__Bacteria; p__Proteobacteria; c__Gammaproteobacteria; o__Cardiobacteriales; f__Cardiobacteriaceae
k__Bacteria; p__Proteobacteria; c__Gammaproteobacteria; o__Chromatiales; f__
k__Bacteria; p__Proteobacteria; c__Gammaproteobacteria; o__Chromatiales; f__Ectothiorhodospiraceae
k__Bacteria; p__Proteobacteria; c__Gammaproteobacteria; o__Enterobacteriales; f__Enterobacteriaceae
k__Bacteria; p__Proteobacteria; c__Gammaproteobacteria; o__Legionellales; f__Coxiellaceae
k__Bacteria; p__Proteobacteria; c__Gammaproteobacteria; o__Methylococcales; f__
k__Bacteria; p__Proteobacteria; c__Gammaproteobacteria; o__Methylococcales; f__Methylococcaceae
k__Bacteria; p__Proteobacteria; c__Gammaproteobacteria; o__Oceanospirillales; f__Halomonadaceae
k__Bacteria; p__Proteobacteria; c__Gammaproteobacteria; o__Oceanospirillales; f__Oceanospirillaceae
k__Bacteria; p__Proteobacteria; c__Gammaproteobacteria; o__Pasteurellales; f__Pasteurellaceae
k__Bacteria; p__Proteobacteria; c__Gammaproteobacteria; o__Pseudomonadales; f__Moraxellaceae
k__Bacteria; p__Proteobacteria; c__Gammaproteobacteria; o__Pseudomonadales; f__Pseudomonadaceae
k__Bacteria; p__Proteobacteria; c__Gammaproteobacteria; o__Thiotrichales; f__Piscirickettsiaceae
k__Bacteria; p__Proteobacteria; c__Gammaproteobacteria; o__Thiotrichales; f__Thiotrichaceae
k__Bacteria; p__Proteobacteria; c__Gammaproteobacteria; o__Vibrionales; f__Vibrionaceae
k__Bacteria; p__Proteobacteria; c__Gammaproteobacteria; o__Xanthomonadales; f__Sinobacteraceae
k__Bacteria; p__Proteobacteria; c__Gammaproteobacteria; o__Xanthomonadales; f__Xanthomonadaceae
k__Bacteria; p__Proteobacteria; c__Gammaproteobacteria; o__[Marinicellales]; f__[Marinicellaceae]
k__Bacteria; p__Proteobacteria; c__Zetaproteobacteria; o__Mariprofundales; f__Mariprofundaceae
k__Bacteria; p__SAR406; c__AB16; o__Arctic96B-7; f__A714017
k__Bacteria; p__SAR406; c__AB16; o__ZA3648c; f__AEGEAN_185
k__Bacteria; p__Spirochaetes; c__Spirochaetes; o__M2PT2-76; f__
k__Bacteria; p__Spirochaetes; c__Spirochaetes; o__Spirochaetales; f__Spirochaetaceae
k__Bacteria; p__Spirochaetes; c__[Leptospirae]; o__[Leptospirales]; f__Leptospiraceae
k__Bacteria; p__Synergistetes; c__Synergistia; o__Synergistales; f__
k__Bacteria; p__Synergistetes; c__Synergistia; o__Synergistales; f__Synergistaceae
k__Bacteria; p__TM6; c__SBRH58; o__; f__
k__Bacteria; p__TM6; c__SJA-4; o__; f__
k__Bacteria; p__TM7; c__TM7-1; o__; f__
k__Bacteria; p__TM7; c__TM7-3; o__Blgi18; f__
k__Bacteria; p__TM7; c__TM7-3; o__CW040; f__
k__Bacteria; p__TM7; c__TM7-3; o__CW040; f__F16
k__Bacteria; p__TM7; c__TM7-3; o__I025; f__
k__Bacteria; p__TM7; c__TM7-3; o__I025; f__Rs-045
k__Bacteria; p__TPD-58; c__; o__; f__
k__Bacteria; p__Tenericutes; c__CK-1C4-19; o__; f__
k__Bacteria; p__Tenericutes; c__Mollicutes; o__; f__
k__Bacteria; p__Tenericutes; c__Mollicutes; o__Acholeplasmatales; f__Acholeplasmataceae
k__Bacteria; p__Tenericutes; c__Mollicutes; o__Anaeroplasmatales; f__Anaeroplasmataceae
k__Bacteria; p__Tenericutes; c__Mollicutes; o__Entomoplasmatales; f__
k__Bacteria; p__Tenericutes; c__Mollicutes; o__Mycoplasmatales; f__Mycoplasmataceae
k__Bacteria; p__Tenericutes; c__Mollicutes; o__RF39; f__
k__Bacteria; p__Tenericutes; c__RF3; o__ML615J-28; f__
k__Bacteria; p__Verrucomicrobia; c__; o__; f__
k__Bacteria; p__Verrucomicrobia; c__Verrucomicrobiae; o__Verrucomicrobiales; f__Verrucomicrobiaceae
k__Bacteria; p__Verrucomicrobia; c__[Pedosphaerae]; o__; f__
k__Bacteria; p__Verrucomicrobia; c__[Pedosphaerae]; o__[Pedosphaerales]; f__Ellin515
k__Bacteria; p__Verrucomicrobia; c__[Spartobacteria]; o__[Chthoniobacterales]; f__01D2Z36
k__Bacteria; p__WPS-2; c__; o__; f__
k__Bacteria; p__WS1; c__; o__; f__
k__Bacteria; p__WS3; c__PRR-12; o__GN03; f__KSB4
k__Bacteria; p__WS3; c__PRR-12; o__Sediment-1; f__
k__Bacteria; p__ZB3; c__; o__; f__
k__Bacteria; p__[Thermi]; c__Deinococci; o__Deinococcales; f__Deinococcaceae
k__Bacteria; p__[Thermi]; c__Deinococci; o__Deinococcales; f__Trueperaceae
k__Bacteria; p__[Thermi]; c__Deinococci; o__Thermales; f__Thermaceae

C)

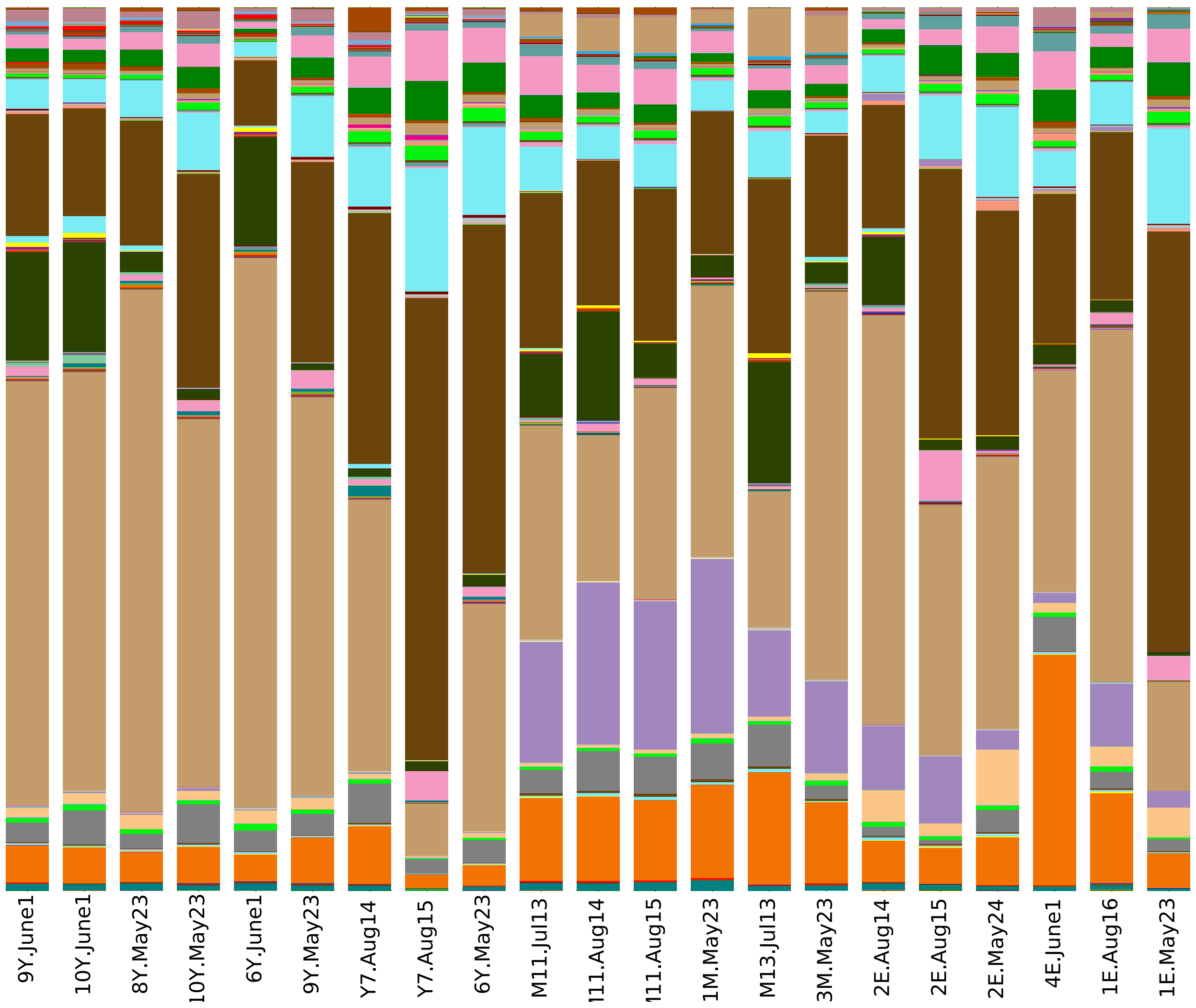
9Y.June1
10Y.June1
8Y.May23
10Y.May23
6Y.June1
9Y.May23
Y7.Aug14
Y7.Aug15
6Y.May23
M11.Jul13
M11.Aug14
M11.Aug15
1M.May23
M13.Jul13
3M.May23
2E.Aug14
2E.Aug15
2E.May24
4E.June1
1E.Aug16
1E.May23

k__Bacteria; p__; c__; o__; f__; g__
k__Bacteria; p__Acidobacteria; c__Acidobacteria-6; o__iii1-15; f__; g__
k__Bacteria; p__Acidobacteria; c__Acidobacteria-6; o__iii1-15; f__RB40; g__
k__Bacteria; p__Acidobacteria; c__Acidobacteria-6; o__iii1-15; f__mb2424; g__
k__Bacteria; p__Acidobacteria; c__Acidobacteriia; o__Acidobacteriales; f__Acidobacteriaceae; g__
k__Bacteria; p__Acidobacteria; c__Acidobacteriia; o__Acidobacteriales; f__Koribacteraceae; g__Candidatus Koribacter
k__Bacteria; p__Acidobacteria; c__BPC102; o__B110; f__; g__
k__Bacteria; p__Acidobacteria; c__Holophagae; o__Holophagales; f__Holophagaceae; g__
k__Bacteria; p__Acidobacteria; c__OS-K; o__; f__; g__
k__Bacteria; p__Acidobacteria; c__RB25; o__; f__; g__
k__Bacteria; p__Acidobacteria; c__Solibacteres; o__Solibacterales; f__; g__
k__Bacteria; p__Acidobacteria; c__Sva0725; o__Sva0725; f__; g__
k__Bacteria; p__Acidobacteria; c__[Chloracidobacteria]; o__PK29; f__; g__
k__Bacteria; p__Acidobacteria; c__[Chloracidobacteria]; o__RB41; f__; g__
k__Bacteria; p__Acidobacteria; c__[Chloracidobacteria]; o__RB41; f__Ellin6075; g__
k__Bacteria; p__Actinobacteria; c__Acidimicrobiia; o__Acidimicrobiales; f__; g__
k__Bacteria; p__Actinobacteria; c__Acidimicrobiia; o__Acidimicrobiales; f__koll13; g__
k__Bacteria; p__Actinobacteria; c__Actinobacteria; o__Actinomycetales; f__Actinomycetaceae; g__Actinomyces
k__Bacteria; p__Actinobacteria; c__Actinobacteria; o__Actinomycetales; f__Actinomycetaceae; g__Varibaculum
k__Bacteria; p__Actinobacteria; c__Actinobacteria; o__Actinomycetales; f__Brevibacteriaceae; g__Brevibacterium
k__Bacteria; p__Actinobacteria; c__Actinobacteria; o__Actinomycetales; f__Cellulomonadaceae; g__
k__Bacteria; p__Actinobacteria; c__Actinobacteria; o__Actinomycetales; f__Corynebacteriaceae; g__Corynebacterium
k__Bacteria; p__Actinobacteria; c__Actinobacteria; o__Actinomycetales; f__Dermabacteraceae; g__Brachybacterium
k__Bacteria; p__Actinobacteria; c__Actinobacteria; o__Actinomycetales; f__Microbacteriaceae; g__
k__Bacteria; p__Actinobacteria; c__Actinobacteria; o__Actinomycetales; f__Microbacteriaceae; g__Microbacterium
k__Bacteria; p__Actinobacteria; c__Actinobacteria; o__Actinomycetales; f__Micrococcaceae; g__
k__Bacteria; p__Actinobacteria; c__Actinobacteria; o__Actinomycetales; f__Mycobacteriaceae; g__Mycobacterium
k__Bacteria; p__Actinobacteria; c__Actinobacteria; o__Actinomycetales; f__Nocardiaceae; g__Rhodococcus
k__Bacteria; p__Actinobacteria; c__Actinobacteria; o__Actinomycetales; f__Pseudonocardiaceae; g__
k__Bacteria; p__Actinobacteria; c__Actinobacteria; o__Actinomycetales; f__Yaniellaceae; g__Yaniella
k__Bacteria; p__Actinobacteria; c__Actinobacteria; o__Bifidobacteriales; f__Bifidobacteriaceae; g__Bifidobacterium
k__Bacteria; p__Actinobacteria; c__Coriobacteriia; o__Coriobacteriales; f__Coriobacteriaceae; g__
k__Bacteria; p__Actinobacteria; c__Coriobacteriia; o__Coriobacteriales; f__Coriobacteriaceae; g__Adlercreutzia
k__Bacteria; p__Actinobacteria; c__Coriobacteriia; o__Coriobacteriales; f__Coriobacteriaceae; g__Collinsella
k__Bacteria; p__Actinobacteria; c__Coriobacteriia; o__Coriobacteriales; f__Coriobacteriaceae; g__Eggerthella
k__Bacteria; p__Actinobacteria; c__Coriobacteriia; o__Coriobacteriales; f__Coriobacteriaceae; g__Slackia
k__Bacteria; p__Actinobacteria; c__MB-A2-108; o__; f__; g__
k__Bacteria; p__Actinobacteria; c__MB-A2-108; o__0319-7L14; f__; g__
k__Bacteria; p__Actinobacteria; c__Nitriliruptoria; o__Nitriliruptorales; f__Nitriliruptoraceae; g__
k__Bacteria; p__Actinobacteria; c__OPB41; o__; f__; g__
k__Bacteria; p__Actinobacteria; c__Rubrobacteria; o__Rubrobacterales; f__Rubrobacteraceae; g__Rubrobacter
k__Bacteria; p__Actinobacteria; c__Thermoleophilia; o__Gaiellales; f__; g__
k__Bacteria; p__Actinobacteria; c__Thermoleophilia; o__Gaiellales; f__Gaiellaceae; g__
k__Bacteria; p__Actinobacteria; c__Thermoleophilia; o__Solirubrobacterales; f__; g__
k__Bacteria; p__Actinobacteria; c__Thermoleophilia; o__Solirubrobacterales; f__Patulibacteraceae; g__
k__Bacteria; p__Armatimonadetes; c__0319-6E2; o__; f__; g__
k__Bacteria; p__Armatimonadetes; c__Chthonomonadetes; o__SJA-22; f__; g__
k__Bacteria; p__Armatimonadetes; c__SJA-176; o__RB046; f__; g__
k__Bacteria; p__Armatimonadetes; c__[Fimbriimonadia]; o__[Fimbriimonadales]; f__[Fimbriimonadaceae]; g__Fimbriimonas
k__Bacteria; p__BHI80-139; c__; o__; f__; g__
k__Bacteria; p__BRC1; c__NPL-UPA2; o__; f__; g__
k__Bacteria; p__BRC1; c__PRR-11; o__; f__; g__
k__Bacteria; p__Bacteroidetes; c__; o__; f__; g__
k__Bacteria; p__Bacteroidetes; c__BME43; o__; f__; g__
k__Bacteria; p__Bacteroidetes; c__Bacteroidia; o__Bacteroidales; f__; g__
k__Bacteria; p__Bacteroidetes; c__Bacteroidia; o__Bacteroidales; f__BA008; g__
k__Bacteria; p__Bacteroidetes; c__Bacteroidia; o__Bacteroidales; f__BS11; g__
k__Bacteria; p__Bacteroidetes; c__Bacteroidia; o__Bacteroidales; f__Bacteroidaceae; g__
k__Bacteria; p__Bacteroidetes; c__Bacteroidia; o__Bacteroidales; f__Bacteroidaceae; g__5-7N15
k__Bacteria; p__Bacteroidetes; c__Bacteroidia; o__Bacteroidales; f__Bacteroidaceae; g__BF311
k__Bacteria; p__Bacteroidetes; c__Bacteroidia; o__Bacteroidales; f__Bacteroidaceae; g__Bacteroides
k__Bacteria; p__Bacteroidetes; c__Bacteroidia; o__Bacteroidales; f__GZKB119; g__
k__Bacteria; p__Bacteroidetes; c__Bacteroidia; o__Bacteroidales; f__ML635J-40; g__
k__Bacteria; p__Bacteroidetes; c__Bacteroidia; o__Bacteroidales; f__Marinilabiaceae; g__
k__Bacteria; p__Bacteroidetes; c__Bacteroidia; o__Bacteroidales; f__Porphyromonadaceae; g__
k__Bacteria; p__Bacteroidetes; c__Bacteroidia; o__Bacteroidales; f__Porphyromonadaceae; g__Candidatus Azobacteroides
k__Bacteria; p__Bacteroidetes; c__Bacteroidia; o__Bacteroidales; f__Porphyromonadaceae; g__Dysgonomonas
k__Bacteria; p__Bacteroidetes; c__Bacteroidia; o__Bacteroidales; f__Porphyromonadaceae; g__Paludibacter
k__Bacteria; p__Bacteroidetes; c__Bacteroidia; o__Bacteroidales; f__Porphyromonadaceae; g__Parabacteroides
k__Bacteria; p__Bacteroidetes; c__Bacteroidia; o__Bacteroidales; f__Porphyromonadaceae; g__Porphyromonas
k__Bacteria; p__Bacteroidetes; c__Bacteroidia; o__Bacteroidales; f__Porphyromonadaceae; g__Tannerella
k__Bacteria; p__Bacteroidetes; c__Bacteroidia; o__Bacteroidales; f__Prevotellaceae; g__
k__Bacteria; p__Bacteroidetes; c__Bacteroidia; o__Bacteroidales; f__Prevotellaceae; g__Prevotella
k__Bacteria; p__Bacteroidetes; c__Bacteroidia; o__Bacteroidales; f__RF16; g__
k__Bacteria; p__Bacteroidetes; c__Bacteroidia; o__Bacteroidales; f__Rikenellaceae; g__
k__Bacteria; p__Bacteroidetes; c__Bacteroidia; o__Bacteroidales; f__Rikenellaceae; g__AF12
k__Bacteria; p__Bacteroidetes; c__Bacteroidia; o__Bacteroidales; f__Rikenellaceae; g__Blvii28
k__Bacteria; p__Bacteroidetes; c__Bacteroidia; o__Bacteroidales; f__Rikenellaceae; g__PW3
k__Bacteria; p__Bacteroidetes; c__Bacteroidia; o__Bacteroidales; f__Rikenellaceae; g__Rikenella
k__Bacteria; p__Bacteroidetes; c__Bacteroidia; o__Bacteroidales; f__S24-7; g__
k__Bacteria; p__Bacteroidetes; c__Bacteroidia; o__Bacteroidales; f__SB-1; g__
k__Bacteria; p__Bacteroidetes; c__Bacteroidia; o__Bacteroidales; f__VC21_Bac22; g__
k__Bacteria; p__Bacteroidetes; c__Bacteroidia; o__Bacteroidales; f__[Barnesiellaceae]; g__
k__Bacteria; p__Bacteroidetes; c__Bacteroidia; o__Bacteroidales; f__[Odoribacteraceae]; g__Butyricimonas
k__Bacteria; p__Bacteroidetes; c__Bacteroidia; o__Bacteroidales; f__[Odoribacteraceae]; g__Odoribacter
k__Bacteria; p__Bacteroidetes; c__Bacteroidia; o__Bacteroidales; f__[Paraprevotellaceae]; g__
k__Bacteria; p__Bacteroidetes; c__Bacteroidia; o__Bacteroidales; f__[Paraprevotellaceae]; g__CF231
k__Bacteria; p__Bacteroidetes; c__Bacteroidia; o__Bacteroidales; f__[Paraprevotellaceae]; g__Paraprevotella
k__Bacteria; p__Bacteroidetes; c__Bacteroidia; o__Bacteroidales; f__[Paraprevotellaceae]; g__YRC22
k__Bacteria; p__Bacteroidetes; c__Bacteroidia; o__Bacteroidales; f__[Paraprevotellaceae]; g__[Prevotella]
k__Bacteria; p__Bacteroidetes; c__Bacteroidia; o__Bacteroidales; f__p-2534-18B5; g__
k__Bacteria; p__Bacteroidetes; c__Cytophagia; o__Cytophagales; f__Cyclobacteriaceae; g__
k__Bacteria; p__Bacteroidetes; c__Cytophagia; o__Cytophagales; f__Cytophagaceae; g__Leadbetterella
k__Bacteria; p__Bacteroidetes; c__Cytophagia; o__Cytophagales; f__Cytophagaceae; g__Spirosoma
k__Bacteria; p__Bacteroidetes; c__Cytophagia; o__Cytophagales; f__Flammeovirgaceae; g__
k__Bacteria; p__Bacteroidetes; c__Cytophagia; o__Cytophagales; f__Flammeovirgaceae; g__Flexibacter
k__Bacteria; p__Bacteroidetes; c__Flavobacteriia; o__; f__; g__
k__Bacteria; p__Bacteroidetes; c__Flavobacteriia; o__Flavobacteriales; f__; g__
k__Bacteria; p__Bacteroidetes; c__Flavobacteriia; o__Flavobacteriales; f__Cryomorphaceae; g__
k__Bacteria; p__Bacteroidetes; c__Flavobacteriia; o__Flavobacteriales; f__Cryomorphaceae; g__Crocinitomix
k__Bacteria; p__Bacteroidetes; c__Flavobacteriia; o__Flavobacteriales; f__Cryomorphaceae; g__Fluviicola
k__Bacteria; p__Bacteroidetes; c__Flavobacteriia; o__Flavobacteriales; f__Flavobacteriaceae; g__
k__Bacteria; p__Bacteroidetes; c__Flavobacteriia; o__Flavobacteriales; f__Flavobacteriaceae; g__Arenibacter
k__Bacteria; p__Bacteroidetes; c__Flavobacteriia; o__Flavobacteriales; f__Flavobacteriaceae; g__Capnocytophaga
k__Bacteria; p__Bacteroidetes; c__Flavobacteriia; o__Flavobacteriales; f__Flavobacteriaceae; g__Flavobacterium
k__Bacteria; p__Bacteroidetes; c__Flavobacteriia; o__Flavobacteriales; f__Flavobacteriaceae; g__Gelidibacter
k__Bacteria; p__Bacteroidetes; c__Flavobacteriia; o__Flavobacteriales; f__Flavobacteriaceae; g__Gillisia
k__Bacteria; p__Bacteroidetes; c__Flavobacteriia; o__Flavobacteriales; f__Flavobacteriaceae; g__Muricauda
k__Bacteria; p__Bacteroidetes; c__Flavobacteriia; o__Flavobacteriales; f__Flavobacteriaceae; g__Polaribacter
k__Bacteria; p__Bacteroidetes; c__Flavobacteriia; o__Flavobacteriales; f__Flavobacteriaceae; g__Psychroflexus
k__Bacteria; p__Bacteroidetes; c__Flavobacteriia; o__Flavobacteriales; f__Flavobacteriaceae; g__Salegentibacter
k__Bacteria; p__Bacteroidetes; c__Flavobacteriia; o__Flavobacteriales; f__NS9; g__
k__Bacteria; p__Bacteroidetes; c__Flavobacteriia; o__Flavobacteriales; f__[Weeksellaceae]; g__
k__Bacteria; p__Bacteroidetes; c__Flavobacteriia; o__Flavobacteriales; f__[Weeksellaceae]; g__Chryseobacterium
k__Bacteria; p__Bacteroidetes; c__Flavobacteriia; o__Flavobacteriales; f__[Weeksellaceae]; g__Cloacibacterium
k__Bacteria; p__Bacteroidetes; c__Sphingobacteriia; o__Sphingobacteriales; f__; g__
k__Bacteria; p__Bacteroidetes; c__Sphingobacteriia; o__Sphingobacteriales; f__Sphingobacteriaceae; g__
k__Bacteria; p__Bacteroidetes; c__Sphingobacteriia; o__Sphingobacteriales; f__Sphingobacteriaceae; g__Pedobacter
k__Bacteria; p__Bacteroidetes; c__Sphingobacteriia; o__Sphingobacteriales; f__Sphingobacteriaceae; g__Sphingobacterium
k__Bacteria; p__Bacteroidetes; c__[Rhodothermi]; o__[Rhodothermales]; f__Rhodothermaceae; g__
k__Bacteria; p__Bacteroidetes; c__[Rhodothermi]; o__[Rhodothermales]; f__Rhodothermaceae; g__Salinibacter
k__Bacteria; p__Bacteroidetes; c__[Rhodothermi]; o__[Rhodothermales]; f__Rhodothermaceae; g__Salisaeta
k__Bacteria; p__Bacteroidetes; c__[Saprospirae]; o__[Saprospirales]; f__; g__
k__Bacteria; p__Bacteroidetes; c__[Saprospirae]; o__[Saprospirales]; f__Chitinophagaceae; g__
k__Bacteria; p__Bacteroidetes; c__[Saprospirae]; o__[Saprospirales]; f__Chitinophagaceae; g__Niabella
k__Bacteria; p__Bacteroidetes; c__[Saprospirae]; o__[Saprospirales]; f__Saprospiraceae; g__
k__Bacteria; p__Caldithrix; c__Caldithrixae; o__Caldithrixales; f__Caldithrixaceae; g__
k__Bacteria; p__Chlorobi; c__; o__; f__; g__
k__Bacteria; p__Chlorobi; c__BSV26; o__A89; f__; g__
k__Bacteria; p__Chlorobi; c__BSV26; o__C20; f__; g__
k__Bacteria; p__Chlorobi; c__BSV26; o__PK329; f__; g__
k__Bacteria; p__Chlorobi; c__Ignavibacteria; o__Ignavibacteriales; f__; g__
k__Bacteria; p__Chlorobi; c__Ignavibacteria; o__Ignavibacteriales; f__Ignavibacteriaceae; g__
k__Bacteria; p__Chlorobi; c__Ignavibacteria; o__Ignavibacteriales; f__[Melioribacteraceae]; g__
k__Bacteria; p__Chlorobi; c__Ignavibacteria; o__Ignavibacteriales; f__lheB3-7; g__
k__Bacteria; p__Chlorobi; c__OPB56; o__; f__; g__
k__Bacteria; p__Chloroflexi; c__Anaerolineae; o__CFB-26; f__; g__
k__Bacteria; p__Chloroflexi; c__Anaerolineae; o__Caldilineales; f__Caldilineaceae; g__
k__Bacteria; p__Chloroflexi; c__Dehalococcoidetes; o__Dehalococcoidales; f__Dehalococcoidaceae; g__
k__Bacteria; p__Chloroflexi; c__S085; o__; f__; g__
k__Bacteria; p__Cyanobacteria; c__4C0d-2; o__SM1D11; f__; g__
k__Bacteria; p__Cyanobacteria; c__4C0d-2; o__YS2; f__; g__
k__Bacteria; p__Cyanobacteria; c__Chloroplast; o__Streptophyta; f__; g__
k__Bacteria; p__Cyanobacteria; c__Gloeobacterophycideae; o__Gloeobacterales; f__Gloeobacteraceae; g__Gloeobacter
k__Bacteria; p__Cyanobacteria; c__Nostocophycideae; o__Stigonematales; f__Rivulariaceae; g__Calothrix
k__Bacteria; p__Cyanobacteria; c__Synechococcophycideae; o__Pseudanabaenales; f__Pseudanabaenaceae; g__
k__Bacteria; p__Cyanobacteria; c__Synechococcophycideae; o__Pseudanabaenales; f__Pseudanabaenaceae; g__Halomicronema
k__Bacteria; p__Cyanobacteria; c__Synechococcophycideae; o__Pseudanabaenales; f__Pseudanabaenaceae; g__Leptolyngbya
k__Bacteria; p__Cyanobacteria; c__Synechococcophycideae; o__Pseudanabaenales; f__Pseudanabaenaceae; g__Pseudanabaena
k__Bacteria; p__Deferribacteres; c__Deferribacteres; o__Deferribacterales; f__; g__
k__Bacteria; p__Deferribacteres; c__Deferribacteres; o__Deferribacterales; f__Deferribacteraceae; g__
k__Bacteria; p__Deferribacteres; c__Deferribacteres; o__Deferribacterales; f__Deferribacteraceae; g__Deferribacter
k__Bacteria; p__Deferribacteres; c__Deferribacteres; o__Deferribacterales; f__Deferribacteraceae; g__Mucispirillum
k__Bacteria; p__Elusimicrobia; c__Elusimicrobia; o__Elusimicrobiales; f__; g__
k__Bacteria; p__FBP; c__; o__; f__; g__
k__Bacteria; p__Fibrobacteres; c__B5-096; o__; f__; g__
k__Bacteria; p__Firmicutes; c__AHT28; o__; f__; g__
k__Bacteria; p__Firmicutes; c__Bacilli; o__Bacillales; f__; g__
k__Bacteria; p__Firmicutes; c__Bacilli; o__Bacillales; f__Alicyclobacillaceae; g__
k__Bacteria; p__Firmicutes; c__Bacilli; o__Bacillales; f__Alicyclobacillaceae; g__Alicyclobacillus
k__Bacteria; p__Firmicutes; c__Bacilli; o__Bacillales; f__Bacillaceae; g__
k__Bacteria; p__Firmicutes; c__Bacilli; o__Bacillales; f__Bacillaceae; g__Alkalibacillus
k__Bacteria; p__Firmicutes; c__Bacilli; o__Bacillales; f__Bacillaceae; g__Anoxybacillus
k__Bacteria; p__Firmicutes; c__Bacilli; o__Bacillales; f__Bacillaceae; g__Bacillus
k__Bacteria; p__Firmicutes; c__Bacilli; o__Bacillales; f__Bacillaceae; g__Marinococcus
k__Bacteria; p__Firmicutes; c__Bacilli; o__Bacillales; f__Bacillaceae; g__Virgibacillus
k__Bacteria; p__Firmicutes; c__Bacilli; o__Bacillales; f__Paenibacillaceae; g__
k__Bacteria; p__Firmicutes; c__Bacilli; o__Bacillales; f__Paenibacillaceae; g__Aneurinibacillus
k__Bacteria; p__Firmicutes; c__Bacilli; o__Bacillales; f__Paenibacillaceae; g__Brevibacillus
k__Bacteria; p__Firmicutes; c__Bacilli; o__Bacillales; f__Paenibacillaceae; g__Cohnella
k__Bacteria; p__Firmicutes; c__Bacilli; o__Bacillales; f__Paenibacillaceae; g__Paenibacillus
k__Bacteria; p__Firmicutes; c__Bacilli; o__Bacillales; f__Planococcaceae; g__
k__Bacteria; p__Firmicutes; c__Bacilli; o__Bacillales; f__Planococcaceae; g__Lysinibacillus
k__Bacteria; p__Firmicutes; c__Bacilli; o__Bacillales; f__Planococcaceae; g__Paenisporosarcina
k__Bacteria; p__Firmicutes; c__Bacilli; o__Bacillales; f__Planococcaceae; g__Planomicrobium
k__Bacteria; p__Firmicutes; c__Bacilli; o__Bacillales; f__Planococcaceae; g__Rummeliibacillus
k__Bacteria; p__Firmicutes; c__Bacilli; o__Bacillales; f__Planococcaceae; g__Solibacillus
k__Bacteria; p__Firmicutes; c__Bacilli; o__Bacillales; f__Planococcaceae; g__Sporosarcina
k__Bacteria; p__Firmicutes; c__Bacilli; o__Bacillales; f__Staphylococcaceae; g__Jeotgalicoccus
k__Bacteria; p__Firmicutes; c__Bacilli; o__Bacillales; f__Staphylococcaceae; g__Salinicoccus
k__Bacteria; p__Firmicutes; c__Bacilli; o__Bacillales; f__Staphylococcaceae; g__Staphylococcus
k__Bacteria; p__Firmicutes; c__Bacilli; o__Bacillales; f__Thermoactinomycetaceae; g__
k__Bacteria; p__Firmicutes; c__Bacilli; o__Bacillales; f__[Exiguobacteraceae]; g__Exiguobacterium
k__Bacteria; p__Firmicutes; c__Bacilli; o__Gemellales; f__; g__
k__Bacteria; p__Firmicutes; c__Bacilli; o__Gemellales; f__Gemellaceae; g__
k__Bacteria; p__Firmicutes; c__Bacilli; o__Gemellales; f__Gemellaceae; g__Gemella
k__Bacteria; p__Firmicutes; c__Bacilli; o__Haloplasmatales; f__Haloplasmataceae; g__
k__Bacteria; p__Firmicutes; c__Bacilli; o__Lactobacillales; f__; g__
k__Bacteria; p__Firmicutes; c__Bacilli; o__Lactobacillales; f__Aerococcaceae; g__
k__Bacteria; p__Firmicutes; c__Bacilli; o__Lactobacillales; f__Aerococcaceae; g__Aerococcus
k__Bacteria; p__Firmicutes; c__Bacilli; o__Lactobacillales; f__Aerococcaceae; g__Alkalibacterium
k__Bacteria; p__Firmicutes; c__Bacilli; o__Lactobacillales; f__Aerococcaceae; g__Alloiococcus
k__Bacteria; p__Firmicutes; c__Bacilli; o__Lactobacillales; f__Aerococcaceae; g__Facklamia
k__Bacteria; p__Firmicutes; c__Bacilli; o__Lactobacillales; f__Carnobacteriaceae; g__
k__Bacteria; p__Firmicutes; c__Bacilli; o__Lactobacillales; f__Carnobacteriaceae; g__Carnobacterium
k__Bacteria; p__Firmicutes; c__Bacilli; o__Lactobacillales; f__Carnobacteriaceae; g__Desemzia
k__Bacteria; p__Firmicutes; c__Bacilli; o__Lactobacillales; f__Carnobacteriaceae; g__Granulicatella
k__Bacteria; p__Firmicutes; c__Bacilli; o__Lactobacillales; f__Carnobacteriaceae; g__Trichococcus
k__Bacteria; p__Firmicutes; c__Bacilli; o__Lactobacillales; f__Enterococcaceae; g__Enterococcus
k__Bacteria; p__Firmicutes; c__Bacilli; o__Lactobacillales; f__Enterococcaceae; g__Vagococcus
k__Bacteria; p__Firmicutes; c__Bacilli; o__Lactobacillales; f__Lactobacillaceae; g__
k__Bacteria; p__Firmicutes; c__Bacilli; o__Lactobacillales; f__Lactobacillaceae; g__Lactobacillus
k__Bacteria; p__Firmicutes; c__Bacilli; o__Lactobacillales; f__Lactobacillaceae; g__Pediococcus
k__Bacteria; p__Firmicutes; c__Bacilli; o__Lactobacillales; f__Leuconostocaceae; g__
k__Bacteria; p__Firmicutes; c__Bacilli; o__Lactobacillales; f__Leuconostocaceae; g__Leuconostoc
k__Bacteria; p__Firmicutes; c__Bacilli; o__Lactobacillales; f__Leuconostocaceae; g__Weissella
k__Bacteria; p__Firmicutes; c__Bacilli; o__Lactobacillales; f__Streptococcaceae; g__
k__Bacteria; p__Firmicutes; c__Bacilli; o__Lactobacillales; f__Streptococcaceae; g__Lactococcus
k__Bacteria; p__Firmicutes; c__Bacilli; o__Lactobacillales; f__Streptococcaceae; g__Streptococcus
k__Bacteria; p__Firmicutes; c__Bacilli; o__Turicibacterales; f__Turicibacteraceae; g__Turicibacter
k__Bacteria; p__Firmicutes; c__Clostridia; o__; f__; g__
k__Bacteria; p__Firmicutes; c__Clostridia; o__BSA2B-08; f__; g__
k__Bacteria; p__Firmicutes; c__Clostridia; o__Clostridiales; f__; g__
k__Bacteria; p__Firmicutes; c__Clostridia; o__Clostridiales; f__Caldicoprobacteraceae; g__Caldicoprobacter
k__Bacteria; p__Firmicutes; c__Clostridia; o__Clostridiales; f__Christensenellaceae; g__
k__Bacteria; p__Firmicutes; c__Clostridia; o__Clostridiales; f__Clostridiaceae; g__
k__Bacteria; p__Firmicutes; c__Clostridia; o__Clostridiales; f__Clostridiaceae; g__02d06
k__Bacteria; p__Firmicutes; c__Clostridia; o__Clostridiales; f__Clostridiaceae; g__Alkaliphilus
k__Bacteria; p__Firmicutes; c__Clostridia; o__Clostridiales; f__Clostridiaceae; g__Caloramator
k__Bacteria; p__Firmicutes; c__Clostridia; o__Clostridiales; f__Clostridiaceae; g__Candidatus Arthromitus
k__Bacteria; p__Firmicutes; c__Clostridia; o__Clostridiales; f__Clostridiaceae; g__Clostridiisalibacter
k__Bacteria; p__Firmicutes; c__Clostridia; o__Clostridiales; f__Clostridiaceae; g__Clostridium
k__Bacteria; p__Firmicutes; c__Clostridia; o__Clostridiales; f__Clostridiaceae; g__Natronincola_Anaerovirgula
k__Bacteria; p__Firmicutes; c__Clostridia; o__Clostridiales; f__Clostridiaceae; g__Oxobacter
k__Bacteria; p__Firmicutes; c__Clostridia; o__Clostridiales; f__Clostridiaceae; g__Proteiniclasticum
k__Bacteria; p__Firmicutes; c__Clostridia; o__Clostridiales; f__Clostridiaceae; g__SMB53
k__Bacteria; p__Firmicutes; c__Clostridia; o__Clostridiales; f__Clostridiaceae; g__Sarcina
k__Bacteria; p__Firmicutes; c__Clostridia; o__Clostridiales; f__Clostridiaceae; g__Tindallia_Anoxynatronum
k__Bacteria; p__Firmicutes; c__Clostridia; o__Clostridiales; f__Dehalobacteriaceae; g__
k__Bacteria; p__Firmicutes; c__Clostridia; o__Clostridiales; f__Dehalobacteriaceae; g__Dehalobacterium
k__Bacteria; p__Firmicutes; c__Clostridia; o__Clostridiales; f__Eubacteriaceae; g__Anaerofustis
k__Bacteria; p__Firmicutes; c__Clostridia; o__Clostridiales; f__Eubacteriaceae; g__Garciella
k__Bacteria; p__Firmicutes; c__Clostridia; o__Clostridiales; f__Eubacteriaceae; g__Pseudoramibacter_Eubacterium
k__Bacteria; p__Firmicutes; c__Clostridia; o__Clostridiales; f__Gracilibacteraceae; g__
k__Bacteria; p__Firmicutes; c__Clostridia; o__Clostridiales; f__Gracilibacteraceae; g__Gracilibacter
k__Bacteria; p__Firmicutes; c__Clostridia; o__Clostridiales; f__Gracilibacteraceae; g__Lutispora
k__Bacteria; p__Firmicutes; c__Clostridia; o__Clostridiales; f__Heliobacteriaceae; g__
k__Bacteria; p__Firmicutes; c__Clostridia; o__Clostridiales; f__Heliobacteriaceae; g__Heliorestis
k__Bacteria; p__Firmicutes; c__Clostridia; o__Clostridiales; f__Lachnospiraceae; g__
k__Bacteria; p__Firmicutes; c__Clostridia; o__Clostridiales; f__Lachnospiraceae; g__Anaerostipes
k__Bacteria; p__Firmicutes; c__Clostridia; o__Clostridiales; f__Lachnospiraceae; g__Blautia
k__Bacteria; p__Firmicutes; c__Clostridia; o__Clostridiales; f__Lachnospiraceae; g__Butyrivibrio
k__Bacteria; p__Firmicutes; c__Clostridia; o__Clostridiales; f__Lachnospiraceae; g__Catonella
k__Bacteria; p__Firmicutes; c__Clostridia; o__Clostridiales; f__Lachnospiraceae; g__Coprococcus
k__Bacteria; p__Firmicutes; c__Clostridia; o__Clostridiales; f__Lachnospiraceae; g__Dorea
k__Bacteria; p__Firmicutes; c__Clostridia; o__Clostridiales; f__Lachnospiraceae; g__Epulopiscium
k__Bacteria; p__Firmicutes; c__Clostridia; o__Clostridiales; f__Lachnospiraceae; g__Lachnobacterium
k__Bacteria; p__Firmicutes; c__Clostridia; o__Clostridiales; f__Lachnospiraceae; g__Lachnospira
k__Bacteria; p__Firmicutes; c__Clostridia; o__Clostridiales; f__Lachnospiraceae; g__Moryella
k__Bacteria; p__Firmicutes; c__Clostridia; o__Clostridiales; f__Lachnospiraceae; g__Oribacterium
k__Bacteria; p__Firmicutes; c__Clostridia; o__Clostridiales; f__Lachnospiraceae; g__Pseudobutyrivibrio
k__Bacteria; p__Firmicutes; c__Clostridia; o__Clostridiales; f__Lachnospiraceae; g__Roseburia
k__Bacteria; p__Firmicutes; c__Clostridia; o__Clostridiales; f__Lachnospiraceae; g__Shuttleworthia
k__Bacteria; p__Firmicutes; c__Clostridia; o__Clostridiales; f__Lachnospiraceae; g__[Ruminococcus]
k__Bacteria; p__Firmicutes; c__Clostridia; o__Clostridiales; f__Peptococcaceae; g__
k__Bacteria; p__Firmicutes; c__Clostridia; o__Clostridiales; f__Peptococcaceae; g__Desulfitobacter
k__Bacteria; p__Firmicutes; c__Clostridia; o__Clostridiales; f__Peptococcaceae; g__Desulfosporosinus
k__Bacteria; p__Firmicutes; c__Clostridia; o__Clostridiales; f__Peptococcaceae; g__Desulfotomaculum
k__Bacteria; p__Firmicutes; c__Clostridia; o__Clostridiales; f__Peptococcaceae; g__Desulfurispora
k__Bacteria; p__Firmicutes; c__Clostridia; o__Clostridiales; f__Peptococcaceae; g__Peptococcus
k__Bacteria; p__Firmicutes; c__Clostridia; o__Clostridiales; f__Peptococcaceae; g__rc4-4
k__Bacteria; p__Firmicutes; c__Clostridia; o__Clostridiales; f__Peptostreptococcaceae; g__
k__Bacteria; p__Firmicutes; c__Clostridia; o__Clostridiales; f__Peptostreptococcaceae; g__Filifactor
k__Bacteria; p__Firmicutes; c__Clostridia; o__Clostridiales; f__Peptostreptococcaceae; g__Peptostreptococcus
k__Bacteria; p__Firmicutes; c__Clostridia; o__Clostridiales; f__Ruminococcaceae; g__
k__Bacteria; p__Firmicutes; c__Clostridia; o__Clostridiales; f__Ruminococcaceae; g__Anaerotruncus
k__Bacteria; p__Firmicutes; c__Clostridia; o__Clostridiales; f__Ruminococcaceae; g__Ethanoligenens
k__Bacteria; p__Firmicutes; c__Clostridia; o__Clostridiales; f__Ruminococcaceae; g__Faecalibacterium
k__Bacteria; p__Firmicutes; c__Clostridia; o__Clostridiales; f__Ruminococcaceae; g__Oscillospira
k__Bacteria; p__Firmicutes; c__Clostridia; o__Clostridiales; f__Ruminococcaceae; g__Ruminococcus
k__Bacteria; p__Firmicutes; c__Clostridia; o__Clostridiales; f__SBYG_4172; g__
k__Bacteria; p__Firmicutes; c__Clostridia; o__Clostridiales; f__Syntrophomonadaceae; g__Syntrophomonas
k__Bacteria; p__Firmicutes; c__Clostridia; o__Clostridiales; f__Veillonellaceae; g__
k__Bacteria; p__Firmicutes; c__Clostridia; o__Clostridiales; f__Veillonellaceae; g__Acidaminococcus
k__Bacteria; p__Firmicutes; c__Clostridia; o__Clostridiales; f__Veillonellaceae; g__BSV43
k__Bacteria; p__Firmicutes; c__Clostridia; o__Clostridiales; f__Veillonellaceae; g__Dialister
k__Bacteria; p__Firmicutes; c__Clostridia; o__Clostridiales; f__Veillonellaceae; g__Megasphaera
k__Bacteria; p__Firmicutes; c__Clostridia; o__Clostridiales; f__Veillonellaceae; g__Pelosinus
k__Bacteria; p__Firmicutes; c__Clostridia; o__Clostridiales; f__Veillonellaceae; g__Phascolarctobacterium
k__Bacteria; p__Firmicutes; c__Clostridia; o__Clostridiales; f__Veillonellaceae; g__Selenomonas
k__Bacteria; p__Firmicutes; c__Clostridia; o__Clostridiales; f__Veillonellaceae; g__Sporomusa
k__Bacteria; p__Firmicutes; c__Clostridia; o__Clostridiales; f__Veillonellaceae; g__Succiniclasticum
k__Bacteria; p__Firmicutes; c__Clostridia; o__Clostridiales; f__Veillonellaceae; g__Thermosinus
k__Bacteria; p__Firmicutes; c__Clostridia; o__Clostridiales; f__Veillonellaceae; g__vadinHB04
k__Bacteria; p__Firmicutes; c__Clostridia; o__Clostridiales; f__[Acidaminobacteraceae]; g__
k__Bacteria; p__Firmicutes; c__Clostridia; o__Clostridiales; f__[Acidaminobacteraceae]; g__Acidaminobacter
k__Bacteria; p__Firmicutes; c__Clostridia; o__Clostridiales; f__[Acidaminobacteraceae]; g__Fusibacter
k__Bacteria; p__Firmicutes; c__Clostridia; o__Clostridiales; f__[Mogibacteriaceae]; g__
k__Bacteria; p__Firmicutes; c__Clostridia; o__Clostridiales; f__[Mogibacteriaceae]; g__Anaerovorax
k__Bacteria; p__Firmicutes; c__Clostridia; o__Clostridiales; f__[Mogibacteriaceae]; g__Mogibacterium
k__Bacteria; p__Firmicutes; c__Clostridia; o__Clostridiales; f__[Tissierellaceae]; g__
k__Bacteria; p__Firmicutes; c__Clostridia; o__Clostridiales; f__[Tissierellaceae]; g__Dethiosulfatibacter
k__Bacteria; p__Firmicutes; c__Clostridia; o__Clostridiales; f__[Tissierellaceae]; g__Finegoldia
k__Bacteria; p__Firmicutes; c__Clostridia; o__Clostridiales; f__[Tissierellaceae]; g__GW-34
k__Bacteria; p__Firmicutes; c__Clostridia; o__Clostridiales; f__[Tissierellaceae]; g__Helcococcus
k__Bacteria; p__Firmicutes; c__Clostridia; o__Clostridiales; f__[Tissierellaceae]; g__Parvimonas
k__Bacteria; p__Firmicutes; c__Clostridia; o__Clostridiales; f__[Tissierellaceae]; g__Peptoniphilus
k__Bacteria; p__Firmicutes; c__Clostridia; o__Clostridiales; f__[Tissierellaceae]; g__Sedimentibacter
k__Bacteria; p__Firmicutes; c__Clostridia; o__Clostridiales; f__[Tissierellaceae]; g__Tepidimicrobium
k__Bacteria; p__Firmicutes; c__Clostridia; o__Clostridiales; f__[Tissierellaceae]; g__ph2
k__Bacteria; p__Firmicutes; c__Clostridia; o__Halanaerobiales; f__; g__
k__Bacteria; p__Firmicutes; c__Clostridia; o__Halanaerobiales; f__Halanaerobiaceae; g__
k__Bacteria; p__Firmicutes; c__Clostridia; o__Halanaerobiales; f__Halobacteroidaceae; g__
k__Bacteria; p__Firmicutes; c__Clostridia; o__MBA08; f__; g__
k__Bacteria; p__Firmicutes; c__Clostridia; o__Natranaerobiales; f__; g__
k__Bacteria; p__Firmicutes; c__Clostridia; o__Natranaerobiales; f__Anaerobrancaceae; g__
k__Bacteria; p__Firmicutes; c__Clostridia; o__Natranaerobiales; f__Anaerobrancaceae; g__Dethiobacter
k__Bacteria; p__Firmicutes; c__Clostridia; o__Natranaerobiales; f__Contubernalisaceae; g__Candidatus Contubernalis
k__Bacteria; p__Firmicutes; c__Clostridia; o__Natranaerobiales; f__ML1228J-1; g__
k__Bacteria; p__Firmicutes; c__Clostridia; o__OPB54; f__; g__
k__Bacteria; p__Firmicutes; c__Clostridia; o__SHA-98; f__; g__
k__Bacteria; p__Firmicutes; c__Clostridia; o__SHA-98; f__D2; g__
k__Bacteria; p__Firmicutes; c__Clostridia; o__Thermoanaerobacterales; f__; g__
k__Bacteria; p__Firmicutes; c__Clostridia; o__Thermoanaerobacterales; f__Caldicellulosiruptoraceae; g__Caldicellulosiruptor
k__Bacteria; p__Firmicutes; c__Clostridia; o__Thermoanaerobacterales; f__Thermoanaerobacteraceae; g__
k__Bacteria; p__Firmicutes; c__Clostridia; o__Thermoanaerobacterales; f__Thermoanaerobacteraceae; g__Moorella
k__Bacteria; p__Firmicutes; c__Clostridia; o__Thermoanaerobacterales; f__Thermoanaerobacteraceae; g__Thermacetogenium
k__Bacteria; p__Firmicutes; c__Clostridia; o__Thermoanaerobacterales; f__Thermodesulfobiaceae; g__Coprothermobacter
k__Bacteria; p__Firmicutes; c__Erysipelotrichi; o__Erysipelotrichales; f__Erysipelotrichaceae; g__
k__Bacteria; p__Firmicutes; c__Erysipelotrichi; o__Erysipelotrichales; f__Erysipelotrichaceae; g__Allobaculum
k__Bacteria; p__Firmicutes; c__Erysipelotrichi; o__Erysipelotrichales; f__Erysipelotrichaceae; g__Bulleidia
k__Bacteria; p__Firmicutes; c__Erysipelotrichi; o__Erysipelotrichales; f__Erysipelotrichaceae; g__Catenibacterium
k__Bacteria; p__Firmicutes; c__Erysipelotrichi; o__Erysipelotrichales; f__Erysipelotrichaceae; g__Coprobacillus
k__Bacteria; p__Firmicutes; c__Erysipelotrichi; o__Erysipelotrichales; f__Erysipelotrichaceae; g__Erysipelothrix
k__Bacteria; p__Firmicutes; c__Erysipelotrichi; o__Erysipelotrichales; f__Erysipelotrichaceae; g__Holdemania
k__Bacteria; p__Firmicutes; c__Erysipelotrichi; o__Erysipelotrichales; f__Erysipelotrichaceae; g__L7A_E11
k__Bacteria; p__Firmicutes; c__Erysipelotrichi; o__Erysipelotrichales; f__Erysipelotrichaceae; g__PSB-M-3
k__Bacteria; p__Firmicutes; c__Erysipelotrichi; o__Erysipelotrichales; f__Erysipelotrichaceae; g__RFN20
k__Bacteria; p__Firmicutes; c__Erysipelotrichi; o__Erysipelotrichales; f__Erysipelotrichaceae; g__[Eubacterium]
k__Bacteria; p__Firmicutes; c__Erysipelotrichi; o__Erysipelotrichales; f__Erysipelotrichaceae; g__cc_115
k__Bacteria; p__Firmicutes; c__Erysipelotrichi; o__Erysipelotrichales; f__Erysipelotrichaceae; g__p-75-a5
k__Bacteria; p__Fusobacteria; c__Fusobacteriia; o__Fusobacteriales; f__Fusobacteriaceae; g__Cetobacterium
k__Bacteria; p__Fusobacteria; c__Fusobacteriia; o__Fusobacteriales; f__Fusobacteriaceae; g__Fusobacterium
k__Bacteria; p__Fusobacteria; c__Fusobacteriia; o__Fusobacteriales; f__Fusobacteriaceae; g__Psychrilyobacter
k__Bacteria; p__GN04; c__MSB-5A5; o__; f__; g__
k__Bacteria; p__GOUTA4; c__; o__; f__; g__
k__Bacteria; p__Gemmatimonadetes; c__; o__; f__; g__
k__Bacteria; p__Gemmatimonadetes; c__Gemm-1; o__; f__; g__
k__Bacteria; p__Gemmatimonadetes; c__Gemm-3; o__; f__; g__
k__Bacteria; p__Gemmatimonadetes; c__JL-ETNP-Z39; o__; f__; g__
k__Bacteria; p__Hyd24-12; c__WM88; o__; f__; g__
k__Bacteria; p__Nitrospirae; c__Nitrospira; o__Nitrospirales; f__0319-6A21; g__
k__Bacteria; p__Nitrospirae; c__Nitrospira; o__Nitrospirales; f__Nitrospiraceae; g__
k__Bacteria; p__OP8; c__; o__; f__; g__
k__Bacteria; p__OP8; c__OP8_1; o__; f__; g__
k__Bacteria; p__Planctomycetes; c__Phycisphaerae; o__Phycisphaerales; f__; g__
k__Bacteria; p__Planctomycetes; c__Planctomycetia; o__Gemmatales; f__Isosphaeraceae; g__
k__Bacteria; p__Planctomycetes; c__Planctomycetia; o__Pirellulales; f__Pirellulaceae; g__
k__Bacteria; p__Proteobacteria; c__; o__; f__; g__
k__Bacteria; p__Proteobacteria; c__Alphaproteobacteria; o__; f__; g__
k__Bacteria; p__Proteobacteria; c__Alphaproteobacteria; o__Caulobacterales; f__; g__
k__Bacteria; p__Proteobacteria; c__Alphaproteobacteria; o__Caulobacterales; f__Caulobacteraceae; g__
k__Bacteria; p__Proteobacteria; c__Alphaproteobacteria; o__Kiloniellales; f__Kiloniellaceae; g__
k__Bacteria; p__Proteobacteria; c__Alphaproteobacteria; o__Rhizobiales; f__; g__
k__Bacteria; p__Proteobacteria; c__Alphaproteobacteria; o__Rhizobiales; f__Aurantimonadaceae; g__
k__Bacteria; p__Proteobacteria; c__Alphaproteobacteria; o__Rhizobiales; f__Hyphomicrobiaceae; g__
k__Bacteria; p__Proteobacteria; c__Alphaproteobacteria; o__Rhizobiales; f__Hyphomicrobiaceae; g__Devosia
k__Bacteria; p__Proteobacteria; c__Alphaproteobacteria; o__Rhizobiales; f__Hyphomicrobiaceae; g__Parvibaculum
k__Bacteria; p__Proteobacteria; c__Alphaproteobacteria; o__Rhizobiales; f__Methylocystaceae; g__
k__Bacteria; p__Proteobacteria; c__Alphaproteobacteria; o__Rhizobiales; f__Rhizobiaceae; g__
k__Bacteria; p__Proteobacteria; c__Alphaproteobacteria; o__Rhizobiales; f__Rhizobiaceae; g__Agrobacterium
k__Bacteria; p__Proteobacteria; c__Alphaproteobacteria; o__Rhizobiales; f__Rhizobiaceae; g__Rhizobium
k__Bacteria; p__Proteobacteria; c__Alphaproteobacteria; o__Rhodobacterales; f__Rhodobacteraceae; g__
k__Bacteria; p__Proteobacteria; c__Alphaproteobacteria; o__Rhodospirillales; f__; g__
k__Bacteria; p__Proteobacteria; c__Alphaproteobacteria; o__Rhodospirillales; f__Acetobacteraceae; g__
k__Bacteria; p__Proteobacteria; c__Alphaproteobacteria; o__Rhodospirillales; f__Rhodospirillaceae; g__
k__Bacteria; p__Proteobacteria; c__Alphaproteobacteria; o__Rickettsiales; f__; g__
k__Bacteria; p__Proteobacteria; c__Alphaproteobacteria; o__Rickettsiales; f__Pelagibacteraceae; g__
k__Bacteria; p__Proteobacteria; c__Alphaproteobacteria; o__Rickettsiales; f__mitochondria; g__
k__Bacteria; p__Proteobacteria; c__Alphaproteobacteria; o__Sphingomonadales; f__Erythrobacteraceae; g__
k__Bacteria; p__Proteobacteria; c__Alphaproteobacteria; o__Sphingomonadales; f__Sphingomonadaceae; g__Kaistobacter
k__Bacteria; p__Proteobacteria; c__Alphaproteobacteria; o__Sphingomonadales; f__Sphingomonadaceae; g__Sphingomonas
k__Bacteria; p__Proteobacteria; c__Betaproteobacteria; o__; f__; g__
k__Bacteria; p__Proteobacteria; c__Betaproteobacteria; o__A21b; f__EB1003; g__
k__Bacteria; p__Proteobacteria; c__Betaproteobacteria; o__Burkholderiales; f__; g__
k__Bacteria; p__Proteobacteria; c__Betaproteobacteria; o__Burkholderiales; f__Alcaligenaceae; g__
k__Bacteria; p__Proteobacteria; c__Betaproteobacteria; o__Burkholderiales; f__Alcaligenaceae; g__Oligella
k__Bacteria; p__Proteobacteria; c__Betaproteobacteria; o__Burkholderiales; f__Alcaligenaceae; g__Sutterella
k__Bacteria; p__Proteobacteria; c__Betaproteobacteria; o__Burkholderiales; f__Burkholderiaceae; g__
k__Bacteria; p__Proteobacteria; c__Betaproteobacteria; o__Burkholderiales; f__Burkholderiaceae; g__Burkholderia
k__Bacteria; p__Proteobacteria; c__Betaproteobacteria; o__Burkholderiales; f__Burkholderiaceae; g__Candidatus Glomeribacter
k__Bacteria; p__Proteobacteria; c__Betaproteobacteria; o__Burkholderiales; f__Burkholderiaceae; g__Salinispora
k__Bacteria; p__Proteobacteria; c__Betaproteobacteria; o__Burkholderiales; f__Comamonadaceae; g__
k__Bacteria; p__Proteobacteria; c__Betaproteobacteria; o__Burkholderiales; f__Comamonadaceae; g__Acidovorax
k__Bacteria; p__Proteobacteria; c__Betaproteobacteria; o__Burkholderiales; f__Comamonadaceae; g__Diaphorobacter
k__Bacteria; p__Proteobacteria; c__Betaproteobacteria; o__Burkholderiales; f__Comamonadaceae; g__Hydrogenophaga
k__Bacteria; p__Proteobacteria; c__Betaproteobacteria; o__Burkholderiales; f__Comamonadaceae; g__Hylemonella
k__Bacteria; p__Proteobacteria; c__Betaproteobacteria; o__Burkholderiales; f__Comamonadaceae; g__Limnobacter
k__Bacteria; p__Proteobacteria; c__Betaproteobacteria; o__Burkholderiales; f__Comamonadaceae; g__Tepidimonas
k__Bacteria; p__Proteobacteria; c__Betaproteobacteria; o__Burkholderiales; f__Oxalobacteraceae; g__
k__Bacteria; p__Proteobacteria; c__Betaproteobacteria; o__Burkholderiales; f__Oxalobacteraceae; g__Cupriavidus
k__Bacteria; p__Proteobacteria; c__Betaproteobacteria; o__Gallionellales; f__Gallionellaceae; g__
k__Bacteria; p__Proteobacteria; c__Betaproteobacteria; o__MND1; f__; g__
k__Bacteria; p__Proteobacteria; c__Betaproteobacteria; o__MWH-UniP1; f__; g__
k__Bacteria; p__Proteobacteria; c__Betaproteobacteria; o__Methylophilales; f__Methylophilaceae; g__Methylotenera
k__Bacteria; p__Proteobacteria; c__Betaproteobacteria; o__Neisseriales; f__Neisseriaceae; g__
k__Bacteria; p__Proteobacteria; c__Betaproteobacteria; o__Neisseriales; f__Neisseriaceae; g__Neisseria
k__Bacteria; p__Proteobacteria; c__Betaproteobacteria; o__Procabacteriales; f__Procabacteriaceae; g__
k__Bacteria; p__Proteobacteria; c__Betaproteobacteria; o__Rhodocyclales; f__Rhodocyclaceae; g__
k__Bacteria; p__Proteobacteria; c__Betaproteobacteria; o__Rhodocyclales; f__Rhodocyclaceae; g__Dechloromonas
k__Bacteria; p__Proteobacteria; c__Betaproteobacteria; o__Rhodocyclales; f__Rhodocyclaceae; g__Dok59
k__Bacteria; p__Proteobacteria; c__Betaproteobacteria; o__Rhodocyclales; f__Rhodocyclaceae; g__TS34
k__Bacteria; p__Proteobacteria; c__Betaproteobacteria; o__SC-I-84; f__; g__
k__Bacteria; p__Proteobacteria; c__Betaproteobacteria; o__Thiobacterales; f__; g__
k__Bacteria; p__Proteobacteria; c__Deltaproteobacteria; o__Bdellovibrionales; f__Bdellovibrionaceae; g__Bdellovibrio
k__Bacteria; p__Proteobacteria; c__Deltaproteobacteria; o__Desulfarculales; f__Desulfarculaceae; g__
k__Bacteria; p__Proteobacteria; c__Deltaproteobacteria; o__Desulfobacterales; f__Desulfobacteraceae; g__
k__Bacteria; p__Proteobacteria; c__Deltaproteobacteria; o__Desulfobacterales; f__Desulfobacteraceae; g__Desulfococcus
k__Bacteria; p__Proteobacteria; c__Deltaproteobacteria; o__Desulfobacterales; f__Desulfobulbaceae; g__
k__Bacteria; p__Proteobacteria; c__Deltaproteobacteria; o__Desulfovibrionales; f__Desulfovibrionaceae; g__
k__Bacteria; p__Proteobacteria; c__Deltaproteobacteria; o__Desulfovibrionales; f__Desulfovibrionaceae; g__Bilophila
k__Bacteria; p__Proteobacteria; c__Deltaproteobacteria; o__Desulfovibrionales; f__Desulfovibrionaceae; g__Desulfovibrio
k__Bacteria; p__Proteobacteria; c__Deltaproteobacteria; o__Desulfuromonadales; f__Geobacteraceae; g__
k__Bacteria; p__Proteobacteria; c__Deltaproteobacteria; o__IndB3-24; f__; g__
k__Bacteria; p__Proteobacteria; c__Deltaproteobacteria; o__MIZ46; f__; g__
k__Bacteria; p__Proteobacteria; c__Deltaproteobacteria; o__Myxococcales; f__; g__
k__Bacteria; p__Proteobacteria; c__Deltaproteobacteria; o__PB19; f__; g__
k__Bacteria; p__Proteobacteria; c__Deltaproteobacteria; o__Spirobacillales; f__; g__
k__Bacteria; p__Proteobacteria; c__Deltaproteobacteria; o__Syntrophobacterales; f__; g__
k__Bacteria; p__Proteobacteria; c__Deltaproteobacteria; o__Syntrophobacterales; f__Syntrophaceae; g__
k__Bacteria; p__Proteobacteria; c__Deltaproteobacteria; o__Syntrophobacterales; f__Syntrophobacteraceae; g__
k__Bacteria; p__Proteobacteria; c__Deltaproteobacteria; o__Syntrophobacterales; f__Syntrophobacteraceae; g__Syntrophobacter
k__Bacteria; p__Proteobacteria; c__Epsilonproteobacteria; o__Campylobacterales; f__Campylobacteraceae; g__
k__Bacteria; p__Proteobacteria; c__Epsilonproteobacteria; o__Campylobacterales; f__Campylobacteraceae; g__Arcobacter
k__Bacteria; p__Proteobacteria; c__Epsilonproteobacteria; o__Campylobacterales; f__Helicobacteraceae; g__
k__Bacteria; p__Proteobacteria; c__Epsilonproteobacteria; o__Campylobacterales; f__Helicobacteraceae; g__Flexispira
k__Bacteria; p__Proteobacteria; c__Gammaproteobacteria; o__; f__; g__
k__Bacteria; p__Proteobacteria; c__Gammaproteobacteria; o__34P16; f__; g__
k__Bacteria; p__Proteobacteria; c__Gammaproteobacteria; o__Acidithiobacillales; f__Acidithiobacillaceae; g__Acidithiobacillus
k__Bacteria; p__Proteobacteria; c__Gammaproteobacteria; o__Aeromonadales; f__Succinivibrionaceae; g__
k__Bacteria; p__Proteobacteria; c__Gammaproteobacteria; o__Alteromonadales; f__Alteromonadaceae; g__Candidatus Endobugula
k__Bacteria; p__Proteobacteria; c__Gammaproteobacteria; o__Alteromonadales; f__Colwelliaceae; g__
k__Bacteria; p__Proteobacteria; c__Gammaproteobacteria; o__Alteromonadales; f__Shewanellaceae; g__Shewanella
k__Bacteria; p__Proteobacteria; c__Gammaproteobacteria; o__Cardiobacteriales; f__; g__
k__Bacteria; p__Proteobacteria; c__Gammaproteobacteria; o__Cardiobacteriales; f__Cardiobacteriaceae; g__
k__Bacteria; p__Proteobacteria; c__Gammaproteobacteria; o__Chromatiales; f__; g__
k__Bacteria; p__Proteobacteria; c__Gammaproteobacteria; o__Chromatiales; f__Ectothiorhodospiraceae; g__
k__Bacteria; p__Proteobacteria; c__Gammaproteobacteria; o__Enterobacteriales; f__Enterobacteriaceae; g__
k__Bacteria; p__Proteobacteria; c__Gammaproteobacteria; o__Enterobacteriales; f__Enterobacteriaceae; g__Proteus
k__Bacteria; p__Proteobacteria; c__Gammaproteobacteria; o__Enterobacteriales; f__Enterobacteriaceae; g__Serratia
k__Bacteria; p__Proteobacteria; c__Gammaproteobacteria; o__Enterobacteriales; f__Enterobacteriaceae; g__Yersinia
k__Bacteria; p__Proteobacteria; c__Gammaproteobacteria; o__Legionellales; f__Coxiellaceae; g__
k__Bacteria; p__Proteobacteria; c__Gammaproteobacteria; o__Legionellales; f__Coxiellaceae; g__Aquicella
k__Bacteria; p__Proteobacteria; c__Gammaproteobacteria; o__Methylococcales; f__; g__
k__Bacteria; p__Proteobacteria; c__Gammaproteobacteria; o__Methylococcales; f__Methylococcaceae; g__
k__Bacteria; p__Proteobacteria; c__Gammaproteobacteria; o__Oceanospirillales; f__Halomonadaceae; g__Candidatus Portiera
k__Bacteria; p__Proteobacteria; c__Gammaproteobacteria; o__Oceanospirillales; f__Halomonadaceae; g__Halomonas
k__Bacteria; p__Proteobacteria; c__Gammaproteobacteria; o__Oceanospirillales; f__Oceanospirillaceae; g__
k__Bacteria; p__Proteobacteria; c__Gammaproteobacteria; o__Pasteurellales; f__Pasteurellaceae; g__
k__Bacteria; p__Proteobacteria; c__Gammaproteobacteria; o__Pseudomonadales; f__Moraxellaceae; g__
k__Bacteria; p__Proteobacteria; c__Gammaproteobacteria; o__Pseudomonadales; f__Moraxellaceae; g__Acinetobacter
k__Bacteria; p__Proteobacteria; c__Gammaproteobacteria; o__Pseudomonadales; f__Moraxellaceae; g__Moraxella
k__Bacteria; p__Proteobacteria; c__Gammaproteobacteria; o__Pseudomonadales; f__Pseudomonadaceae; g__Pseudomonas
k__Bacteria; p__Proteobacteria; c__Gammaproteobacteria; o__Thiotrichales; f__Piscirickettsiaceae; g__
k__Bacteria; p__Proteobacteria; c__Gammaproteobacteria; o__Thiotrichales; f__Thiotrichaceae; g__
k__Bacteria; p__Proteobacteria; c__Gammaproteobacteria; o__Thiotrichales; f__Thiotrichaceae; g__Thiothrix
k__Bacteria; p__Proteobacteria; c__Gammaproteobacteria; o__Vibrionales; f__Vibrionaceae; g__
k__Bacteria; p__Proteobacteria; c__Gammaproteobacteria; o__Xanthomonadales; f__Sinobacteraceae; g__
k__Bacteria; p__Proteobacteria; c__Gammaproteobacteria; o__Xanthomonadales; f__Sinobacteraceae; g__Steroidobacter
k__Bacteria; p__Proteobacteria; c__Gammaproteobacteria; o__Xanthomonadales; f__Xanthomonadaceae; g__
k__Bacteria; p__Proteobacteria; c__Gammaproteobacteria; o__Xanthomonadales; f__Xanthomonadaceae; g__Arenimonas
k__Bacteria; p__Proteobacteria; c__Gammaproteobacteria; o__Xanthomonadales; f__Xanthomonadaceae; g__Lysobacter
k__Bacteria; p__Proteobacteria; c__Gammaproteobacteria; o__Xanthomonadales; f__Xanthomonadaceae; g__Wohlfahrtiimonas
k__Bacteria; p__Proteobacteria; c__Gammaproteobacteria; o__[Marinicellales]; f__[Marinicellaceae]; g__
k__Bacteria; p__Proteobacteria; c__Zetaproteobacteria; o__Mariprofundales; f__Mariprofundaceae; g__Mariprofundus
k__Bacteria; p__SAR406; c__AB16; o__Arctic96B-7; f__A714017; g__SargSea-WGS
k__Bacteria; p__SAR406; c__AB16; o__ZA3648c; f__AEGEAN_185; g__
k__Bacteria; p__Spirochaetes; c__Spirochaetes; o__M2PT2-76; f__; g__
k__Bacteria; p__Spirochaetes; c__Spirochaetes; o__Spirochaetales; f__Spirochaetaceae; g__
k__Bacteria; p__Spirochaetes; c__[Leptospirae]; o__[Leptospirales]; f__Leptospiraceae; g__Leptospira
k__Bacteria; p__Synergistetes; c__Synergistia; o__Synergistales; f__; g__
k__Bacteria; p__Synergistetes; c__Synergistia; o__Synergistales; f__Synergistaceae; g__Thermoanaerovibrio
k__Bacteria; p__TM6; c__SBRH58; o__; f__; g__
k__Bacteria; p__TM6; c__SJA-4; o__; f__; g__
k__Bacteria; p__TM7; c__TM7-1; o__; f__; g__
k__Bacteria; p__TM7; c__TM7-3; o__Blgi18; f__; g__
k__Bacteria; p__TM7; c__TM7-3; o__CW040; f__; g__
k__Bacteria; p__TM7; c__TM7-3; o__CW040; f__F16; g__
k__Bacteria; p__TM7; c__TM7-3; o__I025; f__; g__
k__Bacteria; p__TM7; c__TM7-3; o__I025; f__Rs-045; g__
k__Bacteria; p__TPD-58; c__; o__; f__; g__
k__Bacteria; p__Tenericutes; c__CK-1C4-19; o__; f__; g__
k__Bacteria; p__Tenericutes; c__Mollicutes; o__; f__; g__
k__Bacteria; p__Tenericutes; c__Mollicutes; o__Acholeplasmatales; f__Acholeplasmataceae; g__Candidatus Phytoplasma
k__Bacteria; p__Tenericutes; c__Mollicutes; o__Anaeroplasmatales; f__Anaeroplasmataceae; g__
k__Bacteria; p__Tenericutes; c__Mollicutes; o__Anaeroplasmatales; f__Anaeroplasmataceae; g__Anaeroplasma
k__Bacteria; p__Tenericutes; c__Mollicutes; o__Entomoplasmatales; f__; g__
k__Bacteria; p__Tenericutes; c__Mollicutes; o__Mycoplasmatales; f__Mycoplasmataceae; g__Mycoplasma
k__Bacteria; p__Tenericutes; c__Mollicutes; o__RF39; f__; g__
k__Bacteria; p__Tenericutes; c__RF3; o__ML615J-28; f__; g__
k__Bacteria; p__Verrucomicrobia; c__; o__; f__; g__
k__Bacteria; p__Verrucomicrobia; c__Verrucomicrobiae; o__Verrucomicrobiales; f__Verrucomicrobiaceae; g__
k__Bacteria; p__Verrucomicrobia; c__Verrucomicrobiae; o__Verrucomicrobiales; f__Verrucomicrobiaceae; g__Akkermansia
k__Bacteria; p__Verrucomicrobia; c__Verrucomicrobiae; o__Verrucomicrobiales; f__Verrucomicrobiaceae; g__MSBL3
k__Bacteria; p__Verrucomicrobia; c__Verrucomicrobiae; o__Verrucomicrobiales; f__Verrucomicrobiaceae; g__Verrucomicrobium
k__Bacteria; p__Verrucomicrobia; c__[Pedosphaerae]; o__; f__; g__
k__Bacteria; p__Verrucomicrobia; c__[Pedosphaerae]; o__[Pedosphaerales]; f__Ellin515; g__
k__Bacteria; p__Verrucomicrobia; c__[Spartobacteria]; o__[Chthoniobacterales]; f__01D2Z36; g__
k__Bacteria; p__WPS-2; c__; o__; f__; g__
k__Bacteria; p__WS1; c__; o__; f__; g__
k__Bacteria; p__WS3; c__PRR-12; o__GN03; f__KSB4; g__
k__Bacteria; p__WS3; c__PRR-12; o__Sediment-1; f__; g__
k__Bacteria; p__ZB3; c__; o__; f__; g__
k__Bacteria; p__[Thermi]; c__Deinococci; o__Deinococcales; f__Deinococcaceae; g__Deinococcus
k__Bacteria; p__[Thermi]; c__Deinococci; o__Deinococcales; f__Trueperaceae; g__GBI-58
k__Bacteria; p__[Thermi]; c__Deinococci; o__Thermales; f__Thermaceae; g__Thermus
